# Synthesis of Disubstituted Carboxonium Derivatives of *Closo*-Decaborate Anion [2,6-B_10_H_8_O_2_CC_6_H_5_]^−^: Theoretical and Experimental Study

**DOI:** 10.3390/molecules28041757

**Published:** 2023-02-13

**Authors:** Ilya N. Klyukin, Anastasia V. Kolbunova, Alexander S. Novikov, Alexey V. Nelyubin, Andrey P. Zhdanov, Alexey S. Kubasov, Nikita A. Selivanov, Alexander Yu. Bykov, Konstantin Yu. Zhizhin, Nikolay T. Kuznetsov

**Affiliations:** 1 Kurnakov Institute of General and Inorganic Chemistry, Russian Academy of Sciences, Leninskii Pr. 31, 117907 Moscow, Russia; 2 Institute of Chemistry, Saint Petersburg State University, Universitetskaya nab. 7–9, 199034 Saint Petersburg, Russia; 3 Research Institute of Chemistry, Peoples’ Friendship University of Russia (RUDN University), Miklukho-Maklaya St. 6, 117198 Moscow, Russia

**Keywords:** *closo*-borates, carboxonium derivatives, reaction mechanism, QTAIM, DFT

## Abstract

A comprehensive study focused on the preparation of disubstituted carboxonium derivatives of *closo*-decaborate anion [2,6-B_10_H_8_O_2_CC_6_H_5_]^−^ was carried out. The proposed synthesis of the target product was based on the interaction between the anion [B_10_H_11_]^−^ and benzoic acid C_6_H_5_COOH. It was shown that the formation of this product proceeds stepwise through the formation of a mono-substituted product [B_10_H_9_OC(OH)C_6_H_5_]^−^. In addition, an alternative one-step approach for obtaining the target derivative is postulated. The structure of tetrabutylammonium salts of carboxonium derivative ((C_4_H_9_)_4_N)[2,6-B_10_H_8_O_2_CC_6_H_5_] was established with the help of X-ray structure analysis. The reaction pathway for the formation of [2,6-B_10_H_8_O_2_CC_6_H_5_]^−^ was investigated with the help of density functional theory (DFT) calculations. This process has an electrophile induced nucleophilic substitution (EINS) mechanism, and intermediate anionic species play a key role. Such intermediates have a structure in which one boron atom coordinates two hydrogen atoms. The regioselectivity for the process of formation for the 2,6-isomer was also proved by theoretical calculations. Generally, in the experimental part, the simple and available approach for producing disubstituted carboxonium derivative was introduced, and the mechanism of this process was investigated with the help of theoretical calculations. The proposed approach can be applicable for the preparation of a wide range of disubstituted derivatives of *closo*-borate anions.

## 1. Introduction

Molecular modelling enables the study of the main features of chemical compounds in a clear and simple way [1,2,3,4,5]. Geometric parameters, chemical bonding, atomic charges, electrostatic potential and many other phenomena can be analyzed quickly and with great accuracy [6,7,8,9,10]. In addition, many methods have been developed for the investigation of the reactivity of molecules and reaction mechanisms [11,12,13]. Utilizing the methods of quantum chemistry, it is possible to investigate potential energy surface and study intermediates and transition states of various reactions [14,15,16]. Methods such as QTAIM (Quantum Theory of Atoms in Molecules), NBO (Natural Bond Orbitals) and ELF (Electron Localization Function) enable the definition of peculiarities in the electronic structure of key intermediates and transition states [17,18,19,20,21]. Conceptual DFT enables an evaluation of the electrophile/nucleophile nature of a chemical system through merely estimating the energy of HOMO/LUMO orbitals [22,23,24,25,26]. Thus, molecular modelling is a powerful tool for examining the chemical transformation of molecules.

All previously mentioned methods and approaches are very applicable for boron cluster compounds such as *closo*-borates, carboranes and metallocarboranes [27,28,29,30]. The chemistry of *closo*-borate anions is wide and diverse [31,32]. Borylated analogues of organic compounds such as esters, nitriles and carboxylic acids play a special role in this field of chemistry [33,34,35,36]. Borylated analogues are more reactive than the original organic molecules [37]. Thus, such borylated systems can interact with a wide range of nucleophiles with the formation of different derivatives of *closo*-borate anions [38]. Derivatives of *closo*-borate anions have found different applications in medicine, catalysis and many other fields [39].

Theoretical investigations of *closo*-borate anions’ reactivity were conducted, using a wide range of methods [40,41,42]. For example, the question “how does the reactivity of boron cluster compounds change depending on their structure?” was addressed using Conceptual DFT [43]. It was found that, with increasing boron cluster size, the nucleophilicity of boron clusters reduced [44]. In addition to the Conceptual DFT approach for some other processes, their mechanisms have been investigated in detail. The main intermediates were identified, and transition states were investigated. For example, the mechanisms of nucleophilic addition reactions to nitrile derivatives of *closo*-decaborate anions [B_n_H_n−1_NCR]^−^ n = 10, 12, R = Alkyl, Aryl were investigated [45,46]. It was demonstrated that the stereoselectivity of the final products depends on the structure of the transition state. The main reason for this phenomena is the formation of intermolecular dihydrogen bond between the hydrogen atom of the boron cluster and the proton from the nucleophile.

The most interesting aspect of the investigation process is EINS process (Electrophile Induced Nucleophilic Substitution) [47,48]. As an electrophile inducer, various Lewis and Bronsted acids (CF_3_COOH, CF_3_SO_3_H, BF_3_*Et_2_O, etc.) can be used [49,50,51]. Different organic molecules such as organic nitriles RCN, ethers R_2_O and thioethers R_2_S can be used as nucleophiles [52,53]. However, despite the widespread occurrence of EINS process in laboratory practice, its mechanism has been studied in only a few papers. The mechanism of chlorination of *closo*-borates with the HCl molecule has been investigated in several publications [54,55]. This paper reports on comprehensive investigations of the key stages of the EINS process. The process of chlorination started with the formation of a complex between anion [B_10_H_10_]^2−^ and the HCl molecule. Then the formation of key intermediate [B_10_H_9_H_2_]^−^ was indicated. This complex undewent the process of elimination with the formation of [B_10_H_9_]^−^ intermediate. The process of nucleophilic addition occurred, and [2-B_10_H_9_Cl]^2−^ was formed. It is particularly noteworthy that regioselectivity of the process was investigated and the preference for substitution at the equatorial position of the boron cluster was proven. In addition, the EINS process was performed on the formation of a carboxylic derivative of the *closo*-borate anion with the general form [B_10_H_9_OCOCH_3_]^2−^ [56]. This process is based on interaction between the [B_10_H_10_]^2−^ anion and acetic acid. At the first stage of the process, a proton from the hydroxy group of acetic acid migrated to the boron cluster cage with the formation of the [B_10_H_11_]^−^ anion. Then the process of H_2_ elimination occurred and the [B_10_H_9_]^−^ intermediate was formed. Finally, this anion species interacted with the CH3COO^−^ anion with formation of B_10_H_9_OCOCH_3_]^2−^.

In the present study, the main focus was on the disubstituted carboxonium derivative [2,6-B_10_H_8_O_2_CC_6_H_5_]^−^. This anion is a member of a class of borylated heterocycles [57]. Previously, derivatives [2,6-B_10_H_8_O_2_CR]^−^ R = CH_3_, C_2_H_5_ were obtained by the interaction between [B_10_H_11_]^−^ and acetic acid CH_3_COOH [58,59]. In this work, based on the same approach, the range of disubstituted carboxonium derivatives was extended and [2,6-B_10_H_8_O_2_CC_6_H_5_]^−^ was obtained. The applied approach is versatile and allows to different carboxonium derivatives to be obtained in a simple way. The key step of target anion formation was analyzed using DFT calculations. The driving force of the 2,6-isomer of disubstituted carboxonium derivative formation was revealed.

## 2. Results and Discussion

### 2.1. Synthesis of [B_10_H_8_O_2_CC_6_H_5_]^−^

Initially, an experimental synthesis of [B_10_H_8_O_2_CC_6_H_5_]^−^ was performed. Synthetic methods are based on analogue protocols for [B_10_H_8_O_2_CCH_3_]^−^ [60], but, during this work, some synthetic protocols were improved. Synthesis of [B_10_H_8_O_2_CCH_3_]^−^ was based on the interaction of [B_10_H_11_]^−^ and acetic acid CH_3_COOH. Acetic acid was chosen as a solvent for synthesis performing. In this case, such a synthetic protocol is not possible because of the high melting temperature of benzoic acid C_6_H_5_COOH. Thus, several approaches for the preparation of this derivative were carried out.

The first synthetic route was based on interactions between [B_10_H_11_]^−^ and C_6_H_5_COOH. This process occurred in a dichloromethane CH_2_Cl_2_ solution at boiling point temperature (41 °C). In the first stage of this process, mono-substituted derivatives of the general form [B_10_H_9_OC(OH)C_6_H_5_]^−^ were obtained. Progress of the process was monitored using a ^11^B NMR spectra. The spectra of mono-substituted derivatives are identical to those of [B_10_H_9_OC(OH)CH_3_]^−^. The signal of one in an apical position lay at 2.3 ppm. The signal from another apical position appeared as a broadband signal in the range of 0.3 to −4.6 ppm. This broadening of the signal can be connected with the intramolecular interaction between a hydrogen atom of the boron cluster and a proton from the hydroxy group of organic moieties. The signal from the substituted position lay at −7.7 ppm. Signals from equatorial boron atoms lay at −23.8, −24.8 and −29.7 ppm. The isolation and purification of mono-substituted derivative is a complicated task, due to its thermal instability. Thus, it is possible to use mono-substituted derivative without additional manipulation; the evaporation of dichloromethane solution and washing with ether are enough.

At the next stage, the heating of mono-substituted derivative [B_10_H_9_OC(OH)C_6_H_5_]^−^ led to intramolecular cyclisation when [B_10_H_8_O_2_CC_6_H_5_]^−^ was obtained. It is noteworthy that the cyclisation process was conducted in the absence of solvent. For this process, a rotary evaporator was used with an air bath oven. ^11^B NMR spectra of [B_10_H_8_O_2_CPh]^−^ are similar to the analogous spectra of [B_10_H_8_O_2_CCH_3_]^−^. A detailed analysis of the ^11^B NMR spectra of [B_10_H_8_O_2_CCH_3_]^−^ was carried out in the authors’ previous work [61]. The ^11^B NMR spectra of [B_10_H_8_O_2_CC_6_H_5_]^−^ had four signals: a signal at 0.0 ppm with integral intensities I = 2, corresponding to the boron atoms from equivalent substituted positions (B2, B6), a signal at −7.1 with integral intensities I = 2, corresponding to boron atoms from equivalent apical positions (B1, B10), signals from equatorial positions appearing at −17.6 (I = 2, B3, B9) and a signal at −30.0 (I = 4, B4, B5, B7, B8).

The process of obtaining [B_10_H_8_O_2_CC_6_H_5_]^−^ can be performed in one stage, without the separation and purification of [B_10_H_9_OC(OH)C_6_H_5_]^−^. In this case, the reaction between [B_10_H_11_]^−^ and C_6_H_5_COOH was carried out in an autoclave at 70 °C. Dichloromethane CH_2_Cl_2_ was also chosen as a solvent. The main advantage of a one-pot preparation of the target disubstituted product is the significant decrease in the total time required for the synthesis, compared with the two-stage approach. The overall yield of the target product is similar to the two-stage route.

**Figure 1:**
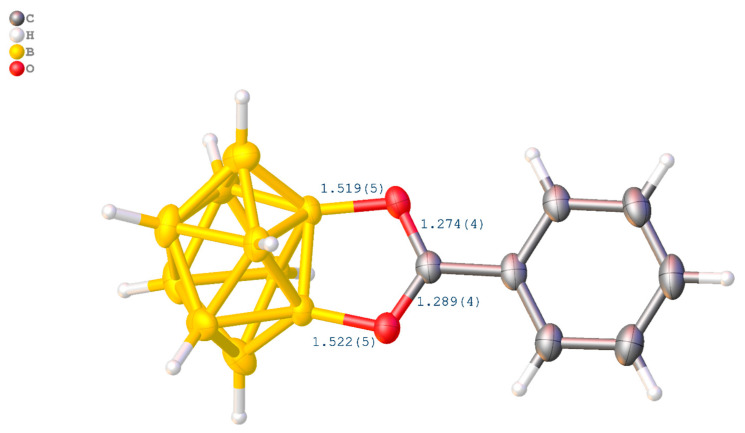
X-ray Structure of [B_10_H_8_O_2_CC_6_H_5_]^−^ anion. Length units—Å.

The crystal structure of ((C_4_H_9_)_4_N)[B_10_H_8_O_2_CC_6_H_5_] salt was established using an X-ray diffraction experiment (Figure 1). The B_2_O_2_C cycle has an almost planar conformation, with torsion angles lying within the interval of 2 to 6°. The B-O bond lengths were in the range of 1.519 to 1.522 Å. The lengths of C-O bonds were quite similar, lying within the interval 1.274 to 1.288 Å. The equality of bond lengths indicates the presence of conjugation in the carboxylic group fragment. The main geometric parameters were quite similar to previously obtained data for the [B_10_H_8_O_2_CCH_3_]^−^ anion. Thus, it can be concluded that the substituent nature of the organic acid fragment had a slight impact on the parameters of B-O and C-O bonds.

**Scheme 1:**
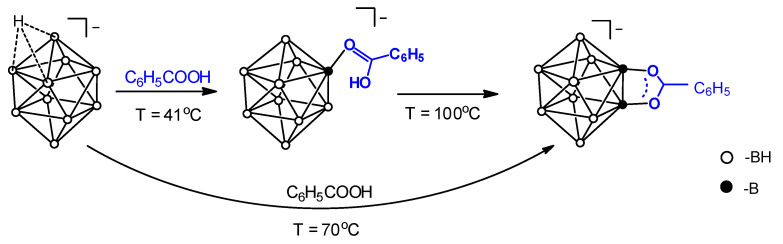
General scheme of preparation of [B_10_H_8_O_2_CC_6_H_5_]^−^ anion.

Several approaches to obtaining the target carboxonium derivative of the general form [B_10_H_8_O_2_CC_6_H_5_]^−^ have been proposed (Scheme 1, for more details also see Scheme S1). It has been shown that the formation of this product proceeds stepwise, through the formation of a mono-substituted product [B_10_H_9_OC(OR)C_6_H_5_]^−^, R = H, C_2_H_5_. Formation of monosubstituted derivative was confirmed with the help of ^11^B NMR spectroscopy. However, it is possible to avoid the mono derivative preparation stage and obtain the target product [B_10_H_8_O_2_CC_6_H_5_]^−^ in one step.

### 2.2. Reaction Mechanism Investigation Based on DFT Calculations

**Figure 2:**
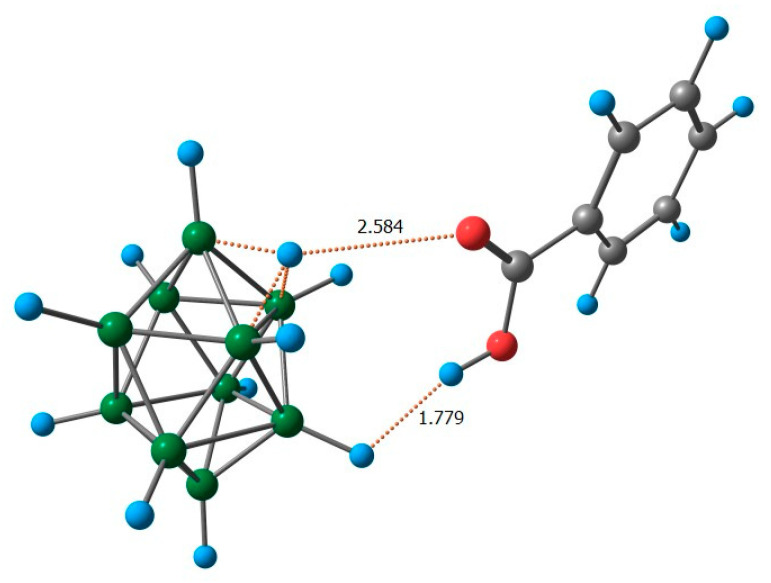
Structure of complex between [B_10_H_11_]^−^ and C_6_H_5_COOH. Length units—Å, ωB97X-D3/def2-TZVPP level of theory.

The process of interaction between [B_10_H_11_]^−^ and C_6_H_5_COOH was studied using DFT calculations. ωB97X-D3 was chosen as DFT functional. The given level of theory is that it is appropriate for a wide range of theoretical calculation issues such as atomic charges, chemical reactivity and covalent and non-covalent interactions [62,63,64]. In addition, the authors successfully used this method for the investigation of closo-borate structures, reactivity and NMR properties [46,61,65]. This mechanism study was conducted to identify the main stages of the process. It is necessary to find out the reason of regioselectivity of formation of disubstituted product. All calculations were carried out with dichlomethane CH_2_Cl_2_ as the solvent. The process of carboxonium derivative formation is related to the EINS process (Electrophile Induced Nucleophilic Substitution). It is well known that the initial step in the EINS process is the elimination of H_2_ from the [B_10_H_11_]^−^ anion, with the formation of [B_10_H_9_]^−^ intermediate. The starting point was the calculation of the orientation complex between [B_10_H_11_]^−^ and C_6_H_5_COOH. The structure of this orientation complex is presented below (Figure 2).

**Figure 3:**
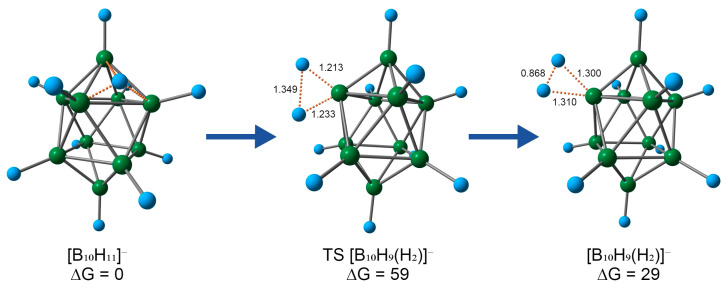
Isomerization process of [B_10_H_11_]^−^ to [B_10_H_9_(H_2_)]^−^. Length units—Å, energy units—kJ/mol, ωB97X-D3/def2-TZVPP level of theory.

However, the formation of the complex between [B_10_H_11_]^−^ and C_6_H_5_COOH is an endergonic process. It can be concluded that, in the first stages of proton migration, the C_6_H_5_COOH molecule does not take part. Thus, initially, H^fac^ migrates to the equatorial boron atom with the formation of a [B_10_H_9_(H_2_)]^−^ anion. The formation of [B_10_H_9_(H_2_)]^−^ occurred through the formation of a transition state. This transition state has a structure in which one boron atom coordinates with dihydrogen, H_2_. The distance between the boron atom and the hydrogen atom was equal to 1.35 Å, the distance between the hydrogen atoms in the H_2_-fragment was equal to 0.86 Å and the energy barrier of the transition state was equal to 59 kJ/mol. The overall process of proton migration was endergonic, and Gibbs energy of the isomerization reaction was 30 kJ/mol (Figure 3). This anion, as in the case of the transition state, had a structure in which one boron atom coordinated with the molecule of dihydrogen, H_2_. The distance between the boron atom and the hydrogen atom was equal to 1.30 Å, while the distance between the hydrogen atoms in the H_2_-fragment was equal to 0.86 Å. Thus, compared with the transition state structure, the B-H bond length increased, and the H-H contact length decreased.

**Figure 4:**
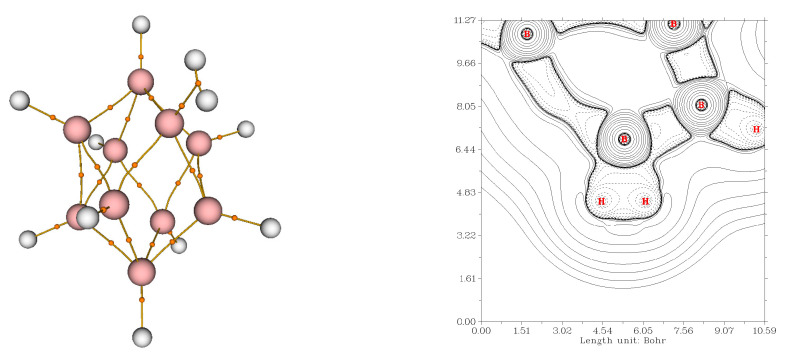
Molecular graph showing the results of the topological analysis of the electron density distribution in the model structure of the [B_10_H_9_(H_2_)]^−^; Contour line map of the Laplacian of electron density for B–(H_2_) fragment, ωB97X-D3/def2-TZVPP level of theory.

The structure of [B_10_H_9_(H_2_)]^−^ was investigated using QTAIM analysis (Figure 4). The bond path between the boron atom and the H_2_ fragment was indicated in the molecular graph of electron density distribution. The main descriptors of electron density at the bond critical point (bcp) corresponding to the interaction between the boron atom and H_2_ were analyzed. The value of *ρ (r)* was equal to 0.126 e Å^−3^, the Laplacian of electron density was equal to 0.350 e Å^−5^, the total energy at the bcp was equal to −0.097 h e^−1^ and the delocalization index was equal to 0.342. In the contour line map of the Laplacian of electron density for the B–(H_2_) fragment, the charge delocalization between boron atom and two hydrogen atoms was observed.

[B_10_H_9_(H_2_)]^−^ underwent the H_2_ elimination process, which is endergonic; Gibbs energy ∆G of this process was 49 kJ/mol. Thus, anion [B_10_H_9_]^−^ was formed. Anion [B_10_H_9_]^−^ is a short-lived intermediate with a vacant orbital on the boron atom. There are two available options of the reaction course. The first one is protonation of the *closo*-borate intermediate with the proton of the hydroxy group of benzoic acid, but this process seems to be unrealistic due to its high endergonicity. Thus, the [B_10_H_9_]^−^ interacts with C_6_H_5_COOH without an activation energy barrier, with the formation of [B_10_H_9_OC(OH)C_6_H_5_]^−^. The process of the formation of the mono-substituted derivative was exergonic, and Gibbs energy of the reaction between [B_10_H_9_]^−^and C_6_H_5_COOH was equal to −118 kJ/mol. The overall exergonic character of the reaction between [B_10_H_9_]^−^ and benzoic acid C_6_H_5_COOH due to the formation of an *exo*-polyhedral B-O bond and the instability of [B_10_H_9_]^−^ being the main driving force of the formation of monosubstituted derivative is favorable.

**Figure 5:**
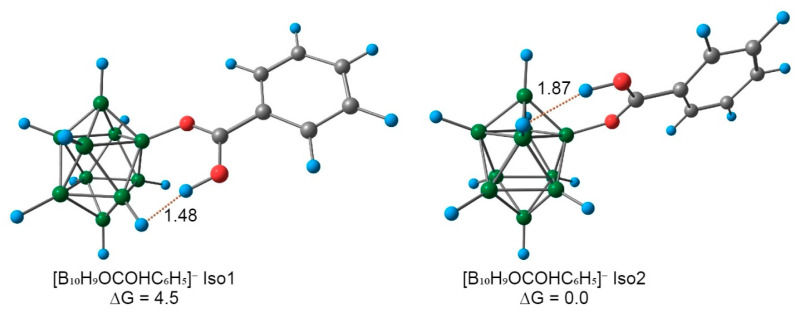
The structure of main isomers of [B_10_H_9_OC(OH)C_6_H_5_]^−^ anion. Length units—Å, energy units—kJ/mol, ωB97X-D3/def2-TZVPP level of theory.

The structure of [B_10_H_9_OC(OH)C_6_H_5_]^−^ was analyzed (Figure 5). Experimentally, the structure of this particle was established only by ^11^B–NMR spectroscopy data. Using theoretical modeling, the data on the structure of this derivative have been supplemented. The organic substituent can rotate freely, relative to the cluster fragment. The rotation barrier of the organic fragment was ~8 kJ/mol. The two lowest energy isomers were localized on the potential energy surface, and the difference in Gibbs energy between them was 3 kJ/mol. The structures of these isomers were stabilized with the formation of an intramolecular dihydrogen bond between the proton of the hydroxy group of the organic substituent and hydrogen from the boron cluster. The B-O bond length was equal to 1.50 Å. The bond length of the C = O bond was equal to 1.25 Å, which was longer than in the case of initial benzoic acid (1.20 Å), whereas the bond length of the C-O bond was equal to 1.29, which was shorter than that of benzoic acid (1.33 Å).

At the next step, the proton from the hydroxy group migrated to the boron cluster. This process occurred without the formation of a transition state. [B_10_H_9_OC(O)C_6_H_5_*H^fac^]^−^ was formed. The process of proton migration from the oxygen atom to the cluster cage was endergonic. As in the case of [B_10_H_9_OC(OH)C_6_H_5_]^−^, the given anion can have different isomers. The proton atom can be localized on different shapes of the boron cluster. The difference between two isomers is less than 1 kJ/mol based on the DFT calculations at the ωB97X-D3/def2-TZVPP level of theory (see Section 3.5. Computational details). Previously, the authors investigated an analogous protonated structure, for [B_10_H_8_O_2_CCH_3_*H^fac^]^−^. H*^fac^* is mainly bonded with apical boron atoms. In the case of [B_10_H_9_OC(O)C_6_H_5_*H^fac^]^−^, the length of H^fac^–B_ap_ was equal to 1.30 Å and H^fac^–B_eq_ was equal to 1.44 to 1.48 Å. Given geometry parameters are also indicate that, in case of [B_10_H_8_O_2_CCH_3_*H^fac^]^−^, H^fac^ was predominantly bonded with the apical boron atom.

**Figure 6:**
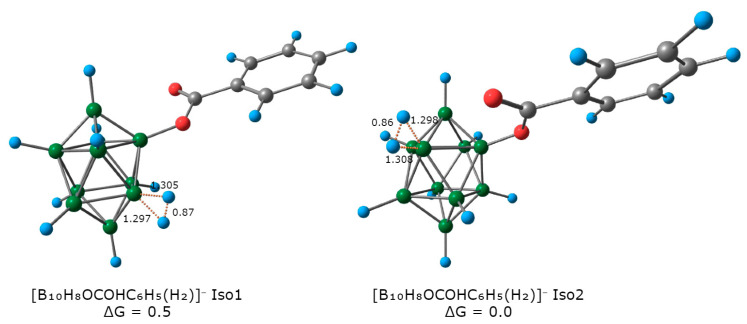
The structure of main isomers of [B_10_H_8_OC(O)C_6_H_5_(H_2_)]^−^ anion. Length units—Å, energy units—kJ/mol, ωB97X-D3/def2-TZVPP level of theory.

H^fac^ migrated with the formation of intermediate [B_10_H_8_OC(O)C_6_H_5_(H_2_)]^−^ (Figure 6). The process of proton migration was endergonic and, as in the case of [B_10_H_9_(H_2_)]^−^, took place through the formation of transition states. The energy barrier of transition states lay within the range 4 to 5 kJ/mol. [B_10_H_8_OC(O)C_6_H_5_(H_2_)]^−^ has several isomers. The structure of two of them is represented below. The structure with the boron atom bonded to H_2_, located in the same equatorial belt as *exo*-polyhedral substituent, had lower Gibbs energy than the structure where the analogous boron atom was located in the opposite equatorial belt to the *exo*-polyhedral substituent. As in the case of [B_10_H_9_(H_2_)]^−^, one boron atom coordinates the molecule of hydrogen, H_2_. The distance between the boron atom and the hydrogen atom was equal to 1.30 Å. The distance between two hydrogen atoms was equal to 0.87 Å.

For [B_10_H_8_OC(O)C_6_H_5_(H_2_)]^−^ systems, QTAIM analysis was performed. As in the case of [B_10_H_9_(H_2_)]^−^, the bond path between the boron atom and the H_2_ fragment was indicated. The main descriptors of the electron density at the bcp were very similar to the descriptors in [B_10_H_9__H_2_]^−^. The value of *ρ (r)* lay in the range of 0.126 to 0.127 e Å^−3^, the Laplacian of electron density was in the range of 0.342 to 0.350 e Å^−5^, total energy at the bcp was equal to −0.098 to −0.099 h e^−1^ and the delocalization index was equal to 0.335 to 0.344. Thus, it can be concluded that the presence of the *exo*-polyhedral substituent had a slight effect on the nature of B-H_2_ interactions.

Then, as in case of [B_10_H_9_(H_2_)]^−^, the H_2_ eliminated. For the [B_10_H_8_OC(O)C_6_H_5_(H_2_)]^−^ isomer, in which the boron atom was bonded to H_2_ in the same equatorial belt as the *exo*-polyhedral substituent, the [B_10_H_8_OC(O)C_6_H_5_]^−^ molecular specie was localized on the potential energy surface. In the case of the isomer, in which the boron atom was in the opposite equatorial belt to the *exo*-polyhedral substituent, [B_10_H_8_OC(O)C_6_H_5_]^−^ was not localized, because the process of geometry optimization led to disubstituted anion [2,6-B_10_H_8_O_2_CC_6_H_5_]^−^.

**Figure 7:**
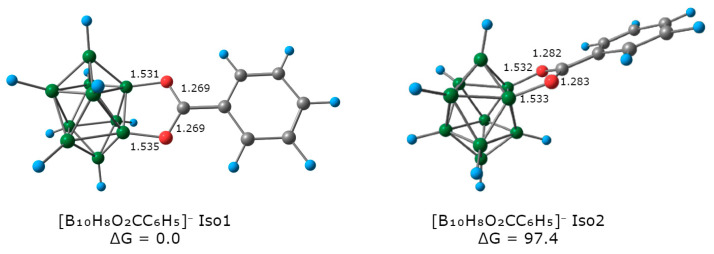
The structure of main isomers of the [B_10_H_8_O_2_CC_6_H_5_]^−^ anion. Length units—Å; energy units—kJ/mol; ωB97X-D3/def2-TZVPP level of theory.

Finally, the process of intramolecular cyclisation occurred, and [B_10_H_8_O_2_CC_6_H_5_]^−^ was formed (Figure 7). This anion had two isomers. The first isomer [2,6-B_10_H_8_O_2_CC_6_H_5_]^−^ had substituted boron atoms in different equatorial belts, whereas the second isomer [2,3-B_10_H_8_O_2_CC_6_H_5_]^−^ had two substituted boron atoms in one equatorial belt. The first isomer [2,6-B_10_H_8_O_2_CC_6_H_5_]^−^ had significantly lower Gibbs energy than the second isomer [2,3-B_10_H_8_O_2_CC_6_H_5_]^−^ (the difference between two isomers is equal to 97.4 kJ/mol). This fact is the reason of high regioselectivity for the formation process of the disubstituted carboxonium derivative. The main geometric parameters of [2,6-B_10_H_8_O_2_CC_6_H_5_]^−^ were considered. The B-O bond lengths were equal to 1.535 Å. The C-O bond lengths were equal to 1.269 Å. Obtained parameters were similar to those previously obtained for the [2,6-B_10_H_8_O_2_CCH_3_]^−^ anion.

**Scheme 2:**
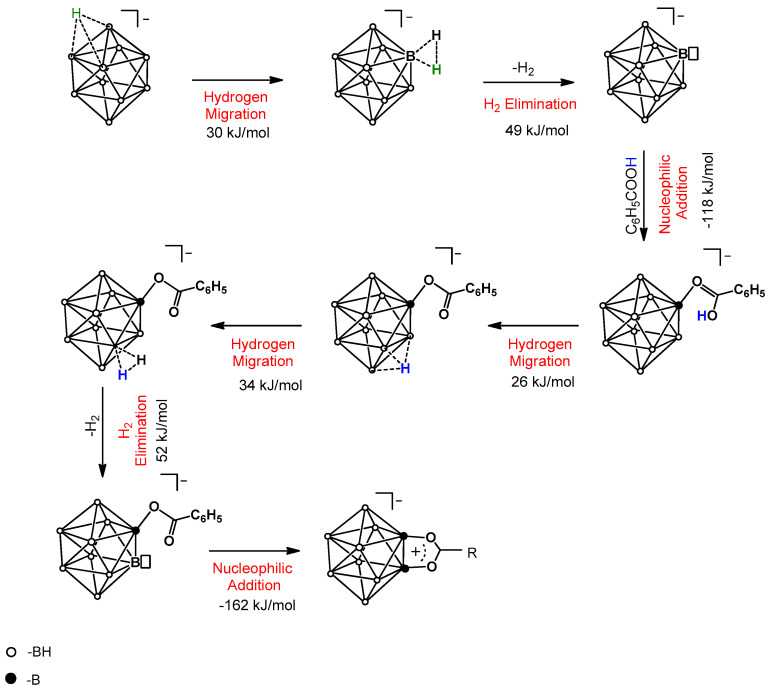
The general mechanism of [B_10_H_8_O_2_CC_6_H_5_]^−^ anion formation.

Thus, the reaction pathway for the formation of [2,6-B_10_H_8_O_2_CC_6_H_5_]^−^ was investigated (Scheme 2). This process was based on the EINS mechanism and, as in the case of a previously described analogous process, the key role was played by intermediate anion species in which one boron atom coordinates two hydrogen atoms. The process of [2,6-B_10_H_8_O_2_CC_6_H_5_]^−^ formation started with hydrogen migration and the formation of [B_10_H_9_(H_2_)]^−^. Then the H_2_ molecule was eliminated, and [B_10_H_9_]^−^ was formed. The process of H_2_ elimination is endergonic, and the ∆G of this process is equal to 49 kJ/mol. Next, [B_10_H_9_]^−^ interacted with C_6_H_5_COOH and mono-substituted derivative [B_10_H_9_OC(OH)C_6_H_5_]^−^ was formed. This process is quite exergonic and the ∆G of this process is −118 kJ/mol. The proton from the hydroxy-group migrated to the boron cage, with the formation of [B_10_H_9_OC(O)C_6_H_5_*H^fac^]^−^. As in the case of [B_10_H_11_]^−^, the proton migrated with the formation of [B_10_H_8_OC(O)C_6_H_5_(H_2_)]^−^. Finally, the H_2_ molecule was eliminated, intermolecular cyclisation occurred and [2,6-B_10_H_8_O_2_CC_6_H_5_]^−^ was formed. The Gibbs energy of the cyclization process was equal to −162 kJ/mol.

## 3. Materials and Methods

### 3.1. IR Spectra

The IR spectra of prepared compounds were recorded on an Infralyum FT 02 Fourier transform spectrometer (Lumex Instruments Research and Production Company, Vancouver, BC, Canada) in the region of 4000 to 300 cm^−1^ and with a resolution of 1 cm^−1^. Samples were prepared as dichloromethane CH_2_Cl_2_ solution.

### 3.2. NMR Spectra

The NMR (^1^H, 11B, 13C) spectra of solutions of the studied compounds in CD_3_CN were recorded on a Bruker (Billerica, MA, USA) Avance II 300 spectrometer operating at 300.3, 96.32 and 75.49 MHz, respectively, using an internal deuterium lock. Tetramethylsilane and Boron trifluoride etherate were used as external references.

### 3.3. Electrospray Ionisation Mass Spectrometry (ESI-MS)

The LC system consisted of two LC-20AD pumps (Shimadzu, Kyoto, Japan), and an autosampler was coupled online with an LCMS-IT-TOF mass spectrometer equipped with an electrospray ionization source (Shimadzu, Kyoto, Japan). The HRMS spectra were acquired in direct injection mode without column. The samples were prepared as CH3CN solutions. Detection parameters: Detector Voltage 1.55 kV; Nebulising Gas 1.50 L/min; CDL Temperature 200.0 °C.

### 3.4. X-ray Crystal Structure Determination

The single-crystal X-ray diffraction data for X were collected on a three-circle Bruker D8 Venture diffractometer using φ and ω scan mode. The data were indexed and integrated using the SAINT program (V7.60A) [66] and then scaled and corrected for absorption using the SADABS program (version 2008/1) [67]. For details, see Table S1. The structures were determined by direct methods and refined by the full-matrix least squares technique on F2 with anisotropic displacement parameters for non-hydrogen atoms. The hydrogen atoms in all compounds were placed in calculated positions and refined within the riding model with fixed isotropic displacement parameters [Uiso(H) = 1.5Ueq(C) for the CH3-groups and 1.2Ueq(C) for the other groups]. All calculations were carried out using the SHELXTL program (version 2018/2) [68] and OLEX2 program package (version 1.5) [69].

Crystallographic data for all investigated compounds have been deposited with the Cambridge Crystallographic Data Centre, CCDC 2,227,408. Copies of this information may be obtained free of charge from the Director, CCDC, 12 Union Road, Cambridge CHB2 1EZ, UK (Fax: +44-1223-336033; e-mail: deposit@ccdc.cam.ac.uk or www.ccdc.cam.ac.uk).

### 3.5. Computational Details

A complete geometry optimization of all model structures was performed at the ωB97X-D3/def2-TZVPP theory level using the ORCA 4.2.1 software package (the atom-pairwise dispersion correction with the zero-damping scheme was utilized) [70,71,72]. All calculations were performed using the RIJCOSX approximation with the def2/J auxiliary basis set [73]. Tight criteria of SCF convergence (“Tight SCF”) were employed for the calculations. The keywords “Grid5”, “FinalGrid6” and “GridX5” were used as parameters for the spatial integration grid. For the model structures with closed electron shells, spin-restricted approximation was utilized. Symmetry operations were not applied during the geometry optimization procedure for all model structures. The Hessian matrices were calculated numerically for all optimized model structures in order to prove the location of correct minima on the potential energy surfaces (no imaginary frequencies for all reactants, intermediates, and final products; only one imaginary frequency for transition states). The connectivity of each reaction step was also confirmed using the intrinsic reaction coordinate (IRC) calculations from the transition states [74,75,76]. Solvent effects were considered using the Solvation Model based on Density (SMD) [77]. The natural bond orbital (NBO) method was employed, using the NBO7 program package [78,79]. Topological analysis of the electron density distribution, based on the Quantum Theory of Atoms in Molecules (QTAIM) formalism developed by Bader [80,81], was employed with the Multiwfn program (version 3.7) [82]. The Cartesian atomic coordinates for all optimized equilibrium model structures are presented in the Supplementary Materials as the xyz-files. Visualization of the optimized structures was carried out with the help of ChemCraft program version 1.7 [83].

### 3.6. Materials

Solvents of reagent and special purity grades Sigma-Aldrich (Burlington, MA, USA) and Panreac (Darmstadt, Germany) (99.7%), were used without any additional purification.

### 3.7. Synthesis of ((C_4_H_9_)_4_N)[2,6-B_10_H_8_O_2_CC_6_H_5_]

Method A: ((C_4_H_9_)_4_N)[B_10_H_11_] (0.250 g, 0.69 mmol) and C_6_H_5_COOH (550 mg, 4.5 mmol) were dissolved in dichloromethane CH_2_Cl_2_ (10 mL). The resulting mixture was heated in an atmosphere of dry argon at 40 to 43 °C for 12 h. After completion of the reaction process, the solution was cooled to room temperature and the solvent was evaporated using a rotary pump vacuum. The residue was washed with Et_2_O (2 × 10 mL) and heated in a vacuum drying oven at 105 °C for 4 h. The product was purified by column chromatography on silica gel, eluting with CHCl_3_/CH_3_CN mixture (9:1). The yield was 0.240 g (74%) ((C_4_H_9_)_4_N)[2,6-B_10_H_8_O_2_CC_6_H_5_]. IR(CH_2_Cl_2_.cm^−1^): 2500 ν(B-H), 1600 ν(C = O). NMR ^11^B-{^1^H} (CD_3_CN, ppm): 0.3 (s B2, B6, I = 2), −6.6 (d, B1, B10, I = 2), −17.1 (d, B3, B9, I = 2), −29.4 (d, B4, B5, B7, B8, I = 4). NMR ^1^H (CD_3_CN, ppm): 7.98 (d, C_6_H_5_, I = 2), 7.73 (t, C_6_H_5_, I = 1), 7.49 (t, C_6_H_5_, I = 2), 3.05 (t, (C_4_H_9_)_4_N^+^, I = 8), 1.57 (m, (C_4_H_9_)_4_N^+^, I = 8), 1.32 (m, (C_4_H_9_)_4_N^+^, I = 8), 0.93 (t, (C_4_H_9_)_4_N^+^, I = 12). NMR ^13^C (CD_3_CN, ppm): 184.5 (O_2_CC_6_H_5_), 136.6 (O_2_CC_6_H_5_), 130.7 (O_2_CC_6_H_5_), 129.3 (O_2_CC_6_H_5_), 124.5 (O_2_CC_6_H_5_), 58.4 ((C_4_H_9_)_4_N^+^), 23.3 ((C_4_H_9_)_4_N^+^), 19.4 ((C_4_H_9_)_4_N^+^), 12.8 ((C_4_H_9_)_4_N^+^). MS (ESI) *m*/*z*: 237.1926 (A refers to the molecular weight of [B_10_H_8_O_2_CC_6_H_5_]. Calculated for {[A]^−^} 237.1924).

Method B: ((C_4_H_9_)_4_N)[B_10_H_11_] (250 mg, 0.69 mmol) and C_6_H_5_COOH (550 mg, 4.5 mmol) were dissolved in dichloromethane CH_2_Cl_2_ (10 mL). The resulting mixture was heated in an autoclave at 75 °C for 4 h. After completion of the reaction process, the solution was cooled to room temperature and the solvent was evaporated using a rotary pump vacuum. The residue was washed with Et_2_O (2 × 10 mL) and purified by column chromatography on silica gel, eluting with CHCl_3_/CH_3_CN mixture (9:1). The yield was 0.255 g (77%) ((C_4_H_9_)_4_N)[2,6-B_10_H_8_O_2_CC_6_H_5_].

## 4. Conclusions

The process of anion [2,6-B_10_H_8_O_2_CC_6_H_5_]^−^ preparation was considered both theoretically and experimentally. This process featured the EINS mechanism. The synthesis of the target product was based on the interaction between [B_10_H_11_]^−^ and benzoic acid C_6_H_5_COOH. The formation of this product proceeded stepwise through the formation of a mono-substituted product [B_10_H_9_OC(OH)C_6_H_5_]^−^. The proposed approach is characterized by a simple apparatus configuration and good yields of final products. This approach can be used for synthesis of disubstituted carboxonium derivative with various natures. Using DFT calculations, the main stages of obtaining [2,6-B_10_H_8_O_2_CC_6_H_5_]^−^ were established. The formation of mono-substituted derivative starts with hydrogen migration and the formation of [B_10_H_9_(H_2_)]^−^. Then the H_2_ molecule eliminates and [B_10_H_9_]^−^ is formed. Next, [B_10_H_9_]^−^ interacts with C_6_H_5_COOH and mono-substituted derivative [B_10_H_9_OC(OH)C_6_H_5_]^−^ is formed. Then the process of disubstituted product [2,6-B_10_H_8_O_2_CC_6_H_5_]^−^ formation takes place. A proton from the hydroxy-group of mono-substituted derivatives migrates to the boron cage with the formation of [B_10_H_9_OC(O)C_6_H_5_*H*^fac^*]^−^. As in the case of [B_10_H_11_]^−^, a proton migrates with the formation of [B_10_H_8_OC(O)C_6_H_5_(H_2_)]^−^. Finally, the H_2_ molecule eliminates, intermolecular cyclisation occurs and [2,6-B_10_H_8_O_2_CC_6_H_5_]^−^ is formed. Based on the modelling results, the reasons for the regioselectivity of the formation of the target product [2,6-B_10_H_8_O_2_CC_6_H_5_]^−^ were established.

## Data Availability

Not applicable.

## Supplementary Materials

The following supporting information can be downloaded at: https://www.mdpi.com/article/10.3390/molecules28041757/s1, Figure S1: ^11^B{^1^H} NMR spectrum of ((C_4_H_9_)_4_N)[2-B_10_H_9_OC(OH)C_6_H_5_]; Figure S2: ^11^B{^1^H} NMR spectrum of ((C_4_H_9_)_4_N)[B_10_H_8_O_2_CC_6_H_5_]. Figure S3: ^1^H NMR spectrum of ((C_4_H_9_)_4_N)[B_10_H_8_O_2_CC_6_H_5_]; Figure S4: ^13^C-NMR spectrum of ((C_4_H_9_)_4_N)[B_10_H_8_O_2_CC_6_H_5_]; Figure S5: ESI-MS spectrum of ((C_4_H_9_)_4_N)[B_10_H_8_O_2_CC_6_H_5_]; Figure S6: IR spectrum of ((C_4_H_9_)_4_N)[B_10_H_8_O_2_CC_6_H_5_]; Table S1: Crystal data and structure refinement for ((C_4_H_9_)_4_N)[B_10_H_8_O_2_CC_6_H_5_]; Scheme S1: Extended scheme of preparation of [B_10_H_8_O_2_CC_6_H_5_]^−^ anion. Figure S7: Contour line map of the electron density for B–(H2) fragment of the [B_10_H_9_(H_2_)]^−^. Figure S8: Molecular graph showing the results of the topological analysis of the electron density distribution in the model structure of the [B_10_H_8_OCOC_6_H_5_(H_2_)]^−^ isomers; Table S2: Main topological parameters of electron density for B–H_2_ interactions; Figure S9: Optimized structure of [B_10_H_9_]^−^ anion. Figure S10: Optimized structure of [B_10_H_8_OCOC_6_H_5_]^−^ anion. Table S3: Cartesian atomic coordinates of the calculated optimized equilibrium model structures.
