# Supplementary material for: Synthesis of Disubstituted Carboxonium Derivatives of Closo-Decaborate Anion [2,6-B10H8O2CC6H5]−: Theoretical and Experimental Study
Source: Molecules. 2023 Feb 13;28(4):1757. doi: 10.3390/molecules28041757 (PMC9966448; doi:10.3390/molecules28041757)

checkCIF/PLATON report

Structure factors have been supplied for datablock(s) cu_22kub245_0m_a

THIS REPORT IS FOR GUIDANCE ONLY. IF USED AS PART OF A REVIEW PROCEDURE FOR PUBLICATION, IT SHOULD NOT REPLACE THE EXPERTISE OF AN EXPERIENCED CRYSTALLOGRAPHIC REFEREE.

No syntax errors found. CIF dictionary Interpreting this report

Datablock: cu_22kub245_0m_a

Bond precision:	C-C = 0.0064 A		Wavelength=1.54178
Cell:	a=10.2162(2)	b=15.0449(3)	c=19.1173(3)
	alpha=90	beta=90.438(1)	gamma=90
Temperature:	100 K		

	Calculated	Reported
Volume	2938.28(9)	2938.28(9)
Space group	P 21/n	P 1 21/n 1
Hall group	-P 2yn	-P 2yn
Moiety formula	C7 H13 B10 O2, C16 H36 N	C7 H13 B10 O2, C16 H36 N
Sum formula	C23 H49 B10 N O2	C23 H49 B10 N O2
Mr	479.73	479.73
Dx,g cm-3	1.084	1.084
Z	4	4
Mu (mm-1)	0.449	0.449
F000	1040.0	1040.0
F000’	1042.38	
h,k,lmax	12,17,22	12,17,22
Nref	5206	5172
Tmin,Tmax	0.948,0.978	0.516,0.753
Tmin’	0.935	

Correction method= # Reported T Limits: Tmin=0.516 Tmax=0.753
AbsCorr = MULTI-SCAN

Data completeness= 0.993 Theta(max)= 66.728

R(reflections)= 0.0966(3708) wR2(reflections)= 0.2385(5172)

S = 1.086 Npar= 329

The following ALERTS were generated. Each ALERT has the format
test-name_ALERT_alert-type_alert-level.
Click on the hyperlinks for more details of the test.

Alert level C

```
DIFMX02_ALERT_1_C  The maximum difference density is > 0.1*ZMAX*0.75
            The relevant atom site should be identified.
PLAT094_ALERT_2_C Ratio of Maximum / Minimum Residual Density ....       2.18 Report
PLAT097_ALERT_2_C Large Reported Max.  (Positive) Residual Density       0.67 eA-3
PLAT230_ALERT_2_C Hirshfeld Test Diff for    B4       --B5       .        6.0 s.u.
PLAT340_ALERT_3_C Low Bond Precision on  C-C Bonds ...............    0.00637 Ang.
PLAT906_ALERT_3_C Large K Value in the Analysis of Variance ......     12.922 Check
PLAT906_ALERT_3_C Large K Value in the Analysis of Variance ......      2.920 Check
PLAT911_ALERT_3_C Missing FCF Refl Between Thmin & STh/L=    0.596         34 Report
```

Alert level G

```
PLAT083_ALERT_2_G SHELXL Second Parameter in WGHT  Unusually Large       6.02 Why ?
PLAT767_ALERT_4_G INS Embedded LIST 6 Instruction Should be LIST 4     Please Check
PLAT883_ALERT_1_G No Info/Value for _atom_sites_solution_primary .     Please Do !
PLAT909_ALERT_3_G Percentage of I>2sig(I) Data at Theta(Max) Still        48% Note
PLAT978_ALERT_2_G Number C-C Bonds with Positive Residual Density.          0 Info
```

0 **ALERT level A** = Most likely a serious problem - resolve or explain
0 **ALERT level B** = A potentially serious problem, consider carefully
8 **ALERT level C** = Check. Ensure it is not caused by an omission or oversight
5 **ALERT level G** = General information/check it is not something unexpected

2 ALERT type 1 CIF construction/syntax error, inconsistent or missing data
5 ALERT type 2 Indicator that the structure model may be wrong or deficient
5 ALERT type 3 Indicator that the structure quality may be low
1 ALERT type 4 Improvement, methodology, query or suggestion
0 ALERT type 5 Informative message, check

It is advisable to attempt to resolve as many as possible of the alerts in all categories. Often the minor alerts point to easily fixed oversights, errors and omissions in your CIF or refinement strategy, so attention to these fine details can be worthwhile. In order to resolve some of the more serious problems it may be necessary to carry out additional measurements or structure refinements. However, the purpose of your study may justify the reported deviations and the more serious of these should normally be commented upon in the discussion or experimental section of a paper or in the "special_details" fields of the CIF. checkCIF was carefully designed to identify outliers and unusual parameters, but every test has its limitations and alerts that are not important in a particular case may appear. Conversely, the absence of alerts does not guarantee there are no aspects of the results needing attention. It is up to the individual to critically assess their own results and, if necessary, seek expert advice.

Publication of your CIF in IUCr journals

A basic structural check has been run on your CIF. These basic checks will be run on all CIFs submitted for publication in IUCr journals (*Acta Crystallographica*, *Journal of Applied Crystallography*, *Journal of Synchrotron Radiation*); however, if you intend to submit to *Acta Crystallographica Section C* or *E* or *IUCrData*, you should make sure that full publication checks are run on the final version of your CIF prior to submission.

Publication of your CIF in other journals

Please refer to the *Notes for Authors* of the relevant journal for any special instructions relating to CIF submission.

PLATON version of 12/09/2022; check.def file version of 09/08/2022

Datablock cu_22kub245_0m_a - ellipsoid plot

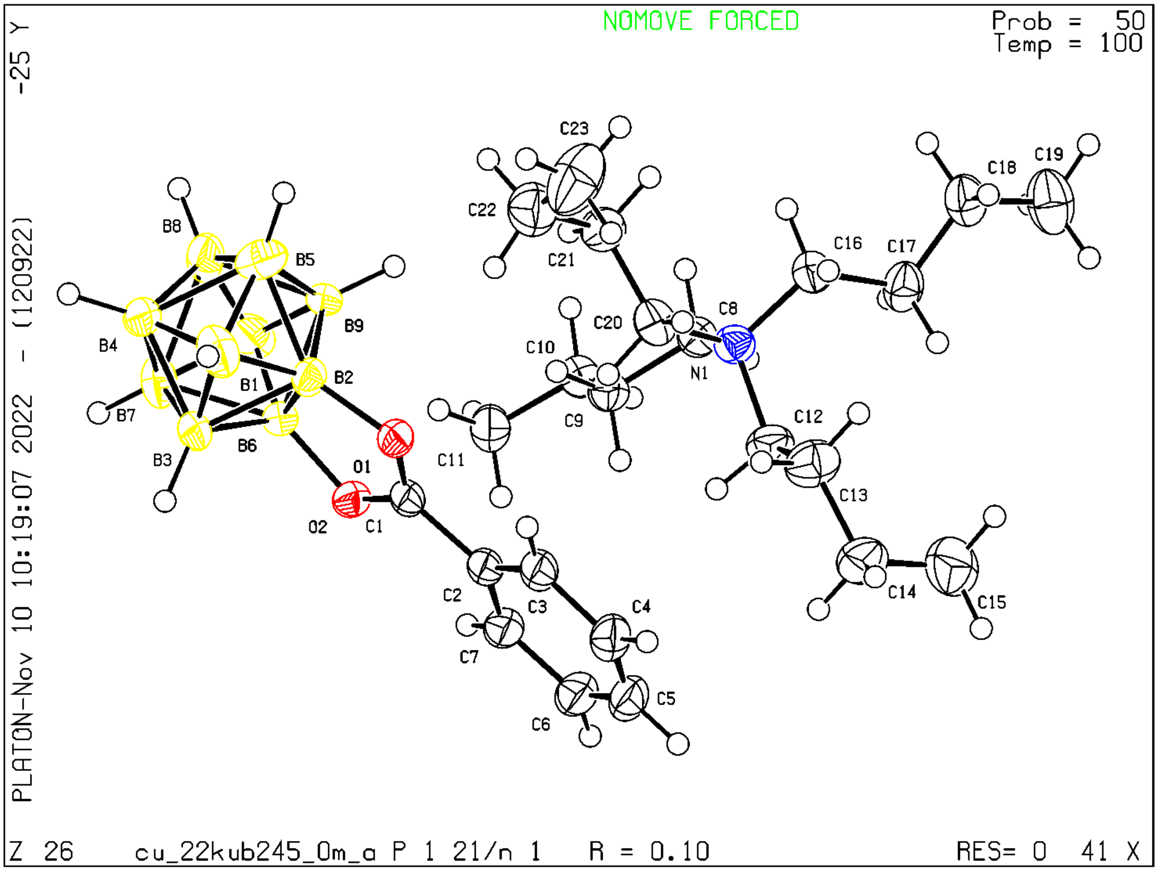

Supplement: Supplementary file 1 [file molecules-28-01757-s001.zip › molecules-2160652-supplementary/Supplementary/B10H8O2CPh_cifreport.pdf]
