# Supplementary material for: Synthesis of Disubstituted Carboxonium Derivatives of Closo-Decaborate Anion [2,6-B10H8O2CC6H5]−: Theoretical and Experimental Study
Source: Molecules. 2023 Feb 13;28(4):1757. doi: 10.3390/molecules28041757 (PMC9966448; doi:10.3390/molecules28041757)
Supplement: Supplementary file 1 [file molecules-28-01757-s001.zip › molecules-2160652-supplementary/Supplementary/Supplementary.pdf]

Supplementary materials

Synthesis of Disubstituted Carboxonium Derivatives of *Closo*-Decaborate Anion $[2,6\text{-}B_{10}H_8O_2CC_6H_5]^-$: Theoretical and Experimental Study

Ilya N. Klyukin [1,*], Anastasia V. Kolbunova [1], Alexander S. Novikov [2,3,*], Alexey V. Nelyubin [1], Andrey P. Zhdanov [1], Alexey S. Kubasov [1], Nikita A. Selivanov [1], Alexander Yu. Bykov [1], Konstantin Yu. Zhizhin [1] and Nikolay T. Kuznetsov [1]

[1] Kurnakov Institute of General and Inorganic Chemistry, Russian Academy of Sciences, Leninskii Pr. 31, 117907 Moscow, Russia
[2] Institute of Chemistry, Saint Petersburg State University, Universitetskaya nab. 7–9, 199034 Saint Petersburg, Russia
[3] Research Institute of Chemistry, Peoples' Friendship University of Russia (RUDN University), Miklukho-Maklaya St. 6, 117198 Moscow, Russia
* Correspondence: klukinil@igic.ras.ru (I.N.K.); a.s.novikov@spbu.ru (A.S.N.)

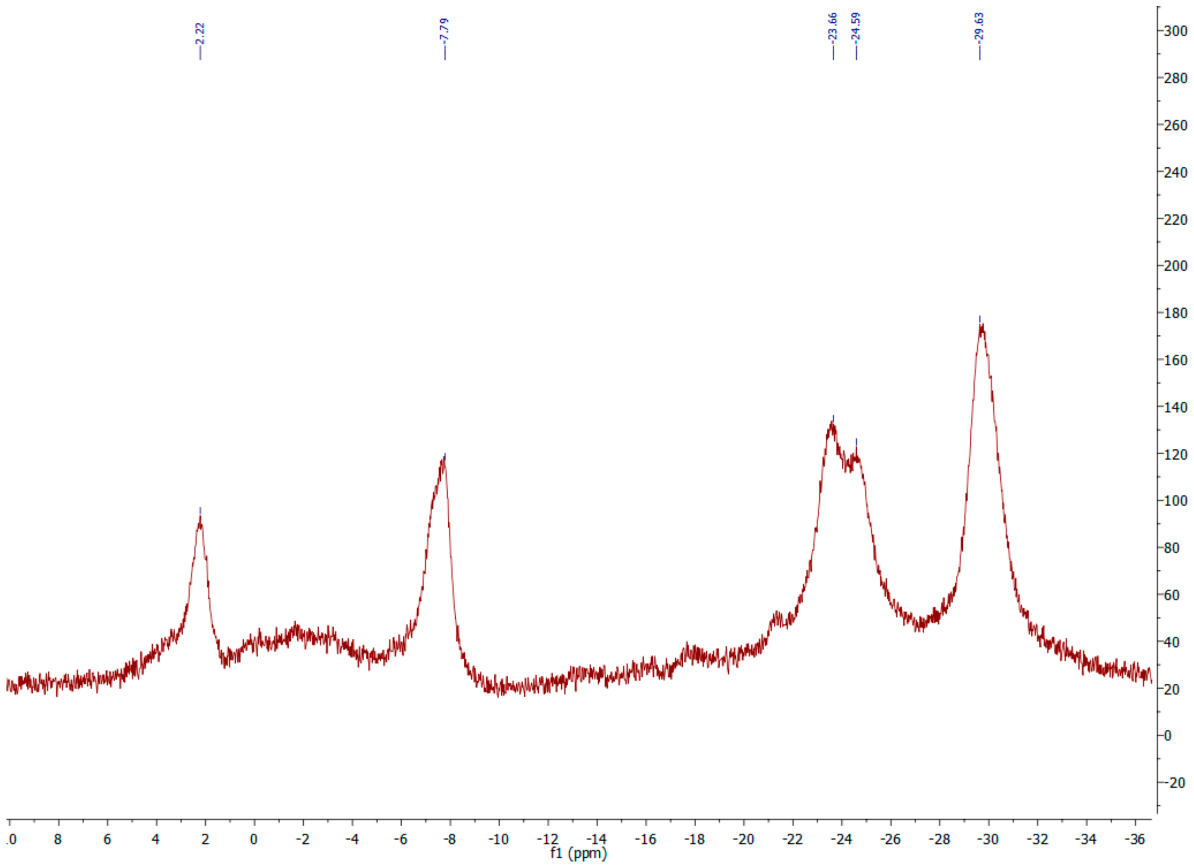

Figure S1. $^{11}B\{^{1}H\}$ NMR spectrum of $((C_4H_9)_4N)[2\text{-}B_{10}H_9OC(OH)C_6H_5]$. Integral intensities of signals were not indicated due it is spectra of reaction mixture.

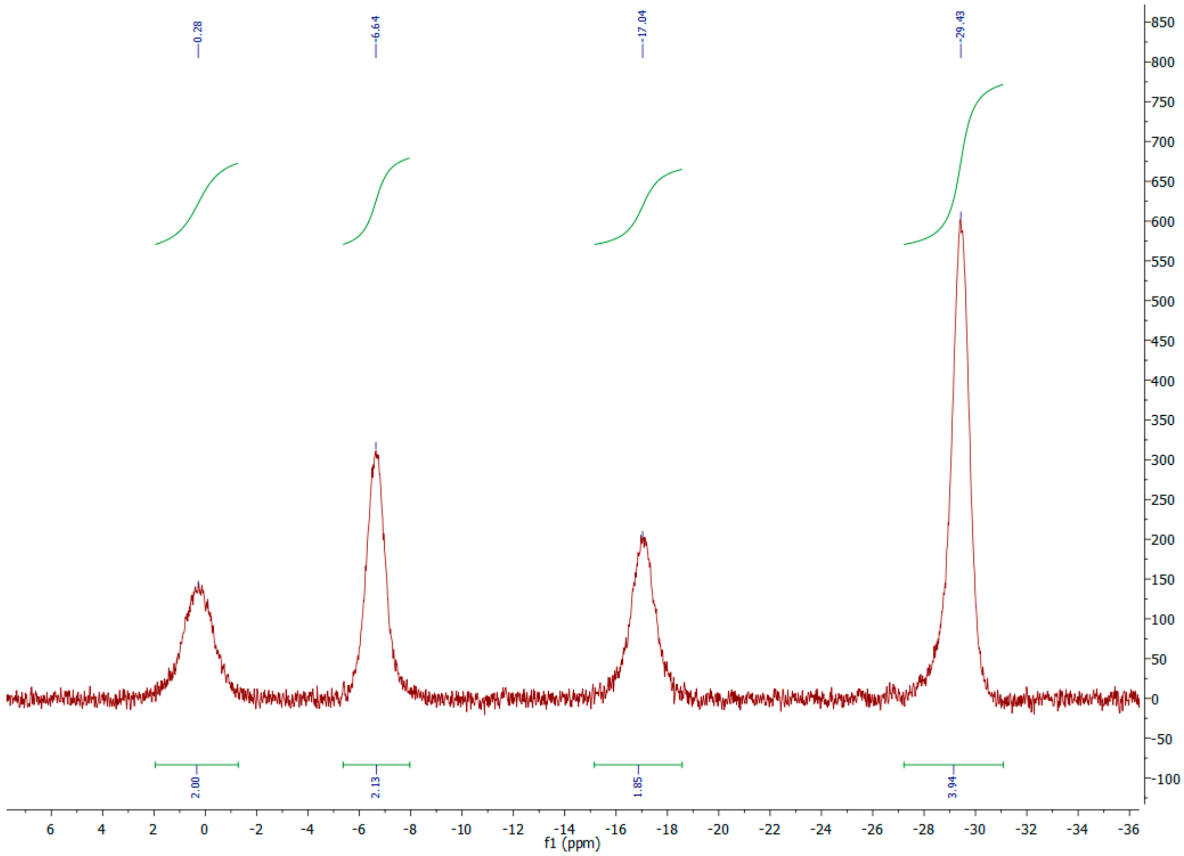

Figure S2. $^{11}B\{^{1}H\}$ NMR spectrum of $((C_4H_9)_4N)[B_{10}H_8O_2CC_6H_5]$.

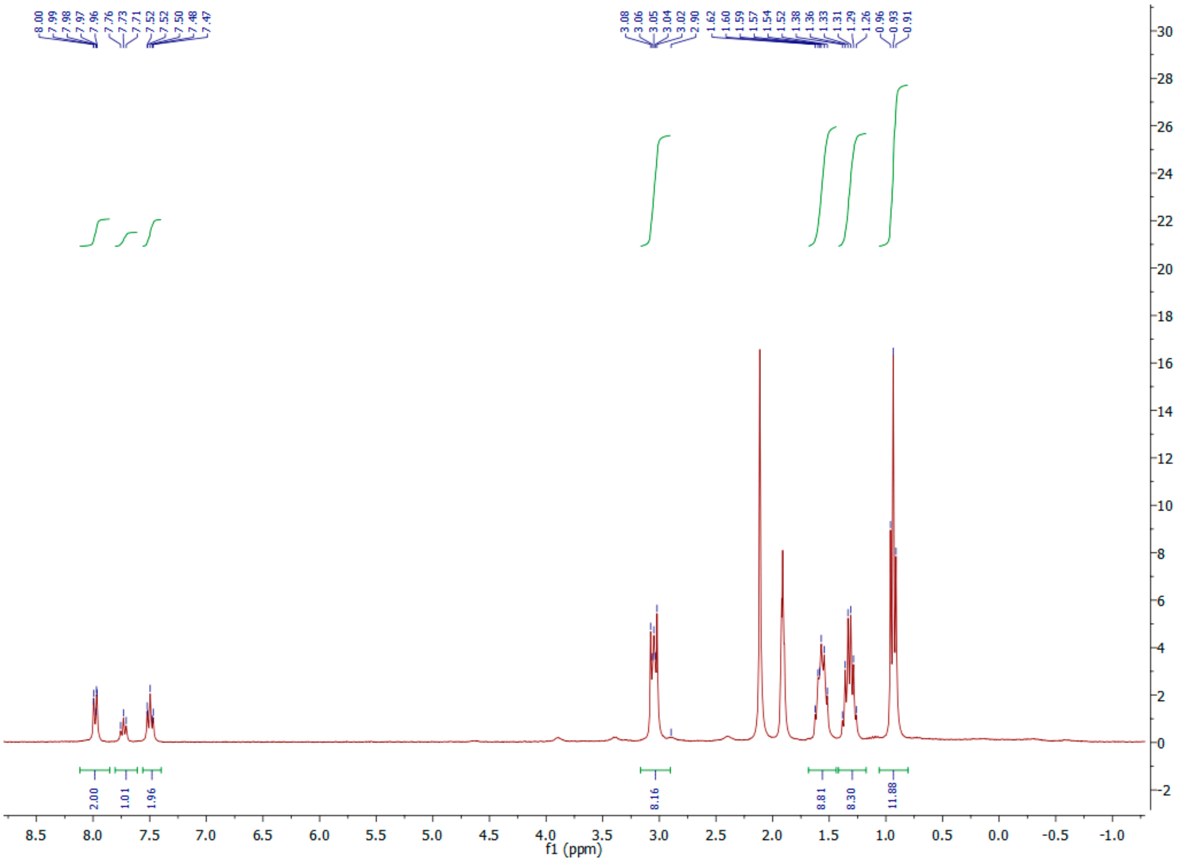

Figure S3. ^{1}H NMR spectrum of $((C_4H_9)_4N)[B_{10}H_8O_2CC_6H_5]$.

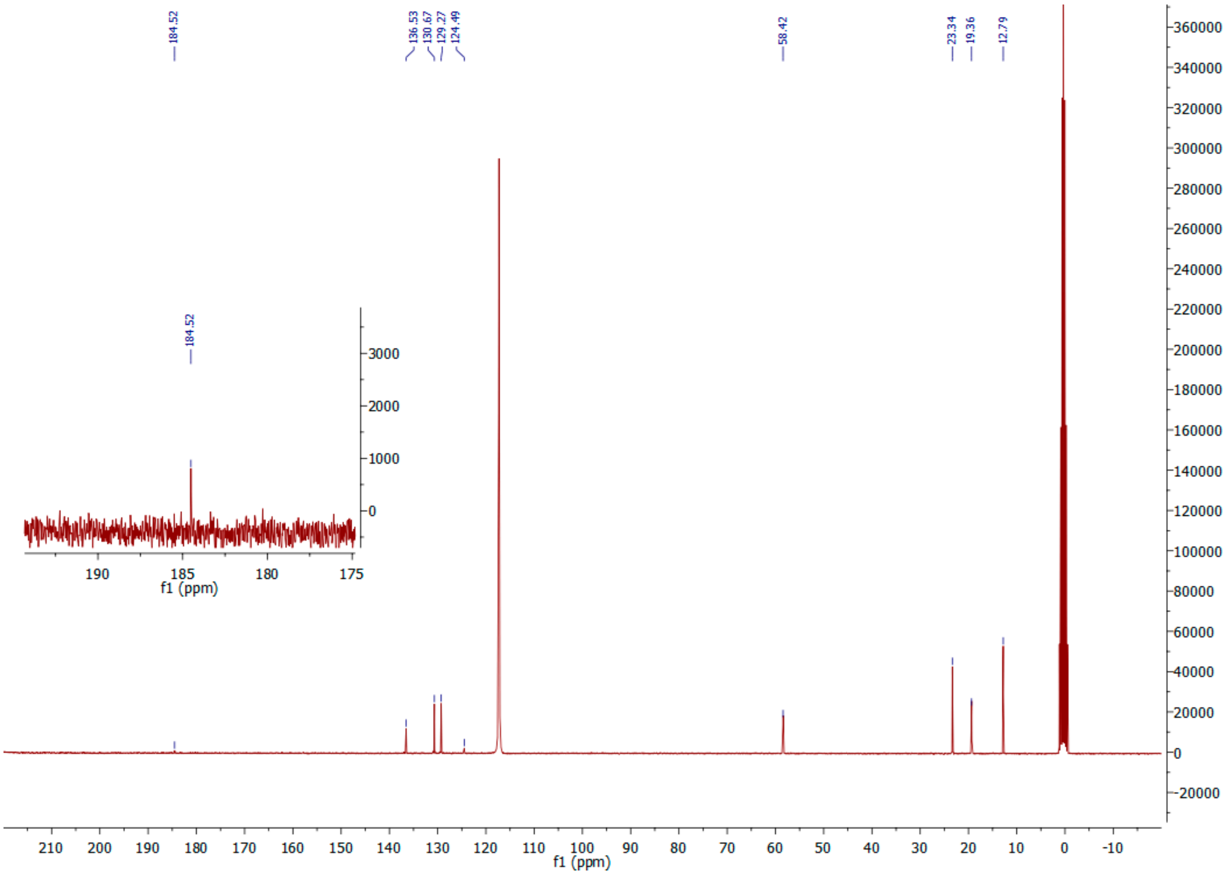

Figure S4. ^{13}C NMR spectrum of $((C_4H_9)_4N)[B_{10}H_8O_2CC_6H_5]$.

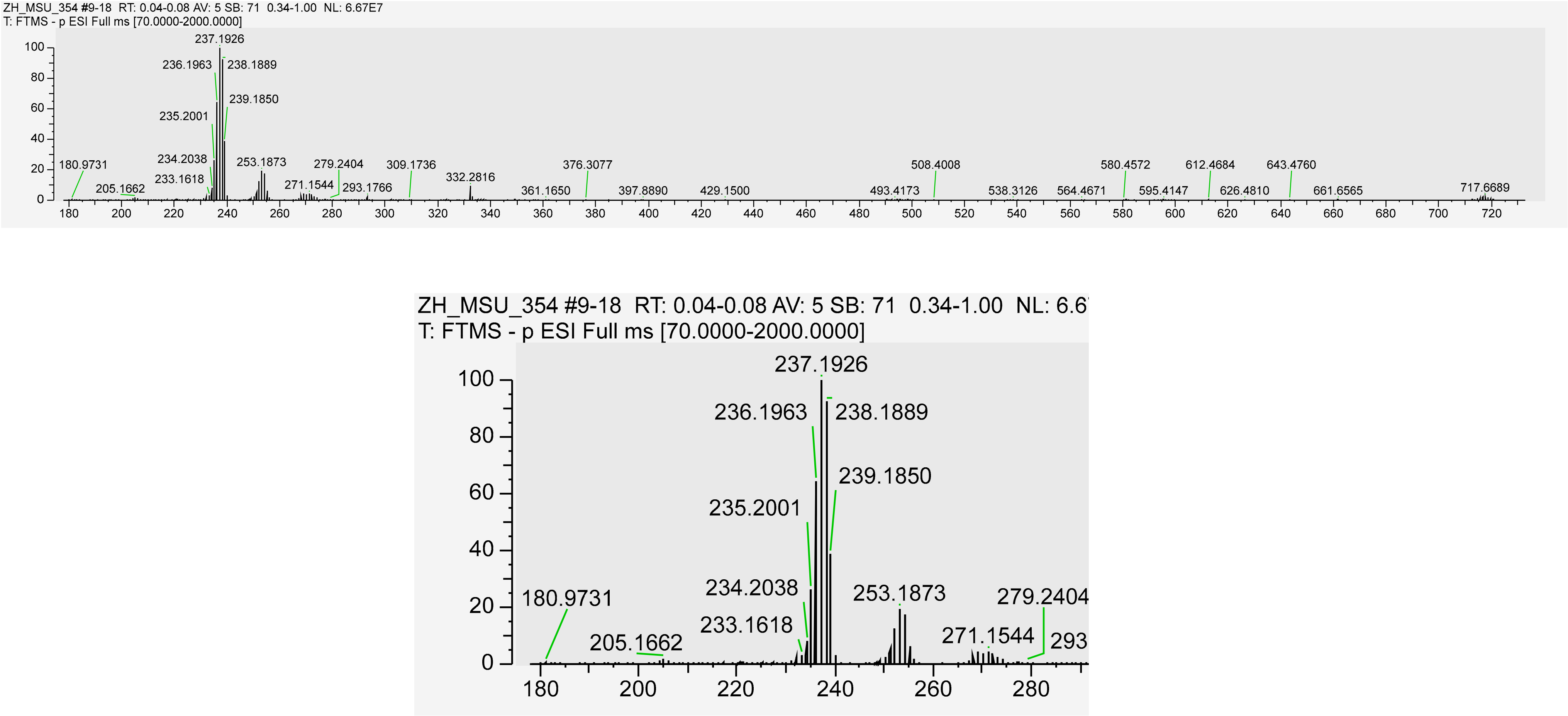

Figure S5. ESI-MS spectrum of $((C_4H_9)_4N)[B_{10}H_8O_2CC_6H_5]$ (full range and zoomed area of molecular ion peaks).

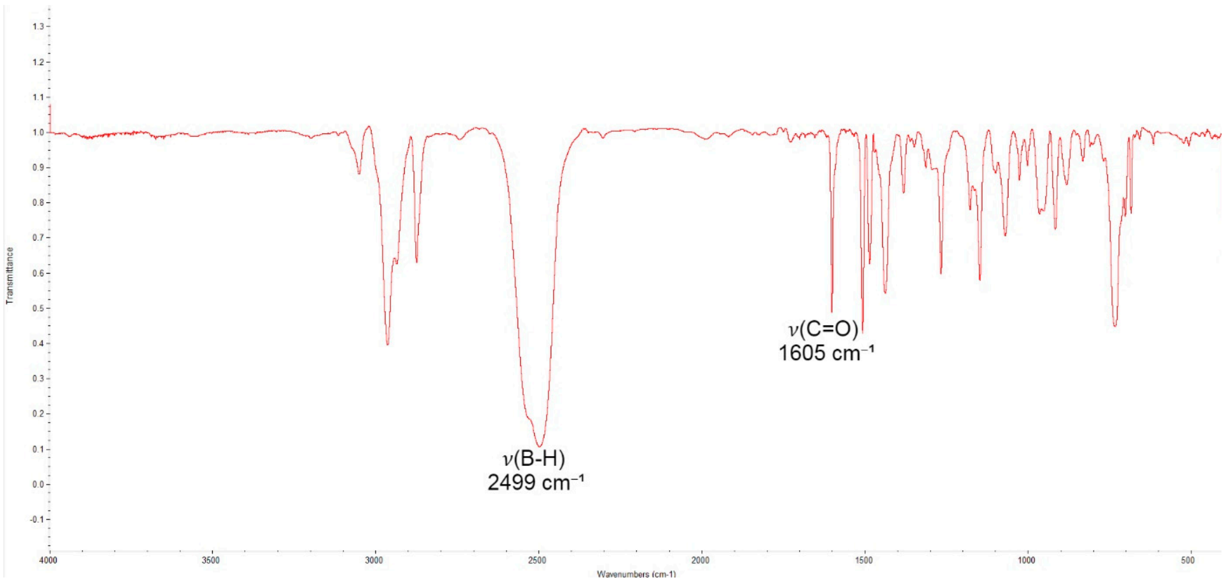

Figure S6. IR spectrum of $((C_4H_9)_4N)[B_{10}H_8O_2CC_6H_5]$.

Table S1. Crystal data and structure refinement for $((C_4H_9)_4N)[B_{10}H_8O_2CC_6H_5]$.

Identification code	$((C_4H_9)_4N)[B_{10}H_8O_2CC_6H_5]^{-}$
Empirical formula	$C_{23}H_{49}B_{10}NO_2$
Formula weight	479.73
Temperature/K	100.00
Crystal system	monoclinic
Space group	$P2_1/n$
a/Å	10.2162(2)
b/Å	15.0449(3)
c/Å	19.1173(3)
β/°	90.4380(10)
Volume/Å^3	2938.28(9)
Z	4
ρ_{calc}g/cm^3	1.084
μ/mm^{-1}	0.449
F(000)	1040.0
Radiation	CuKα (λ = 1.54178)
2Θ range for data collection/°	7.478 to 133.456
Reflections collected	26288
Independent reflections	5172 [R_{int} = 0.0770, R_{sigma} = 0.0586]
Data/restraints/parameters	5172/0/329
Goodness-of-fit on F^2	1.086
Final R indexes [I>=2σ (I)]	R_1 = 0.0966, wR_2 = 0.2187
Final R indexes [all data]	R_1 = 0.1295, wR_2 = 0.2385

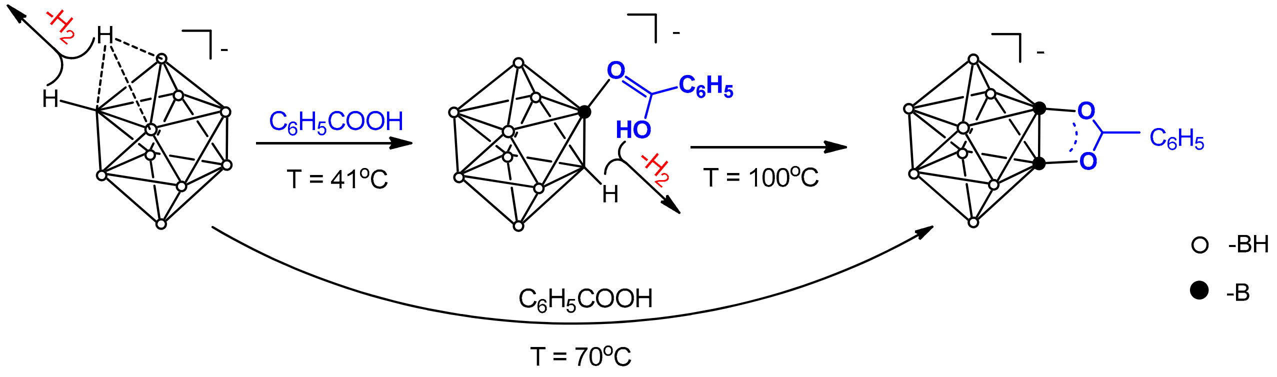

Scheme S1. Extended scheme of preparation of $[B_{10}H_8O_2CC_6H_5]^-$ anion.

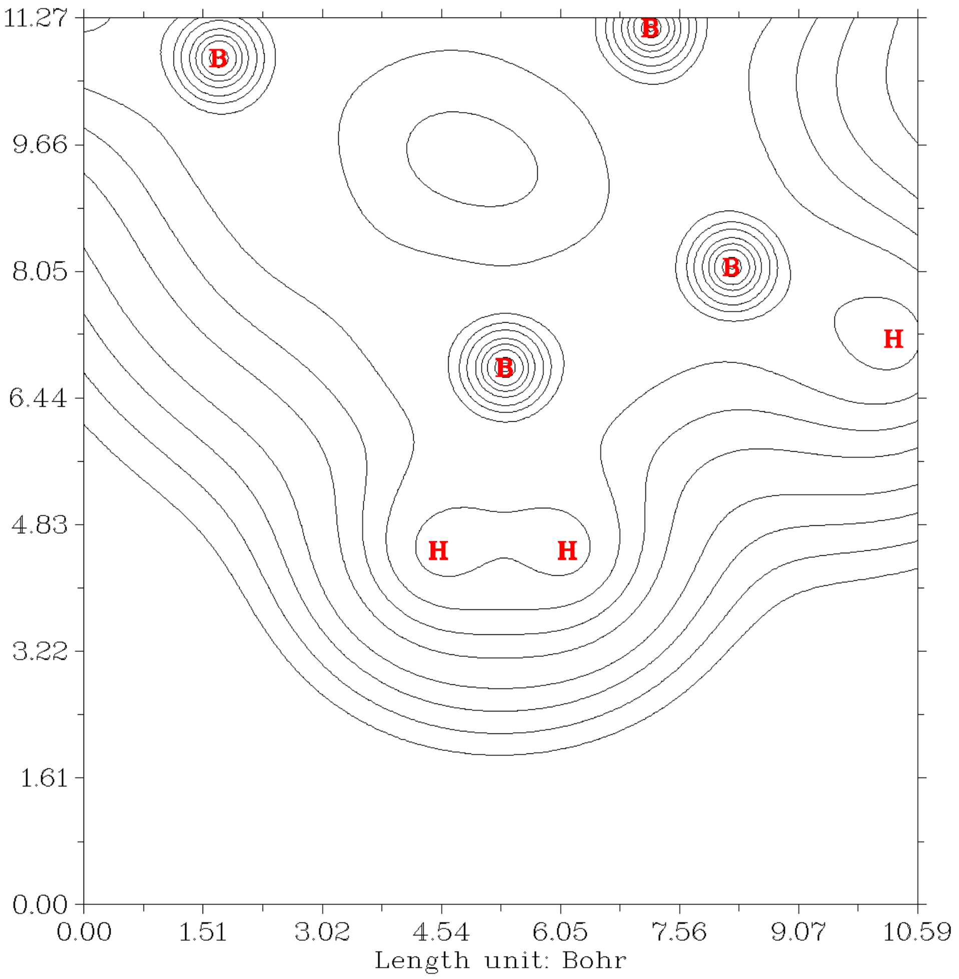

Figure S7. Contour line map of the electron density for B–(H2) fragment of the $[B_{10}H_9(H_2)]^-$, ωB97X-D3/def2-TZVPP level of theory.

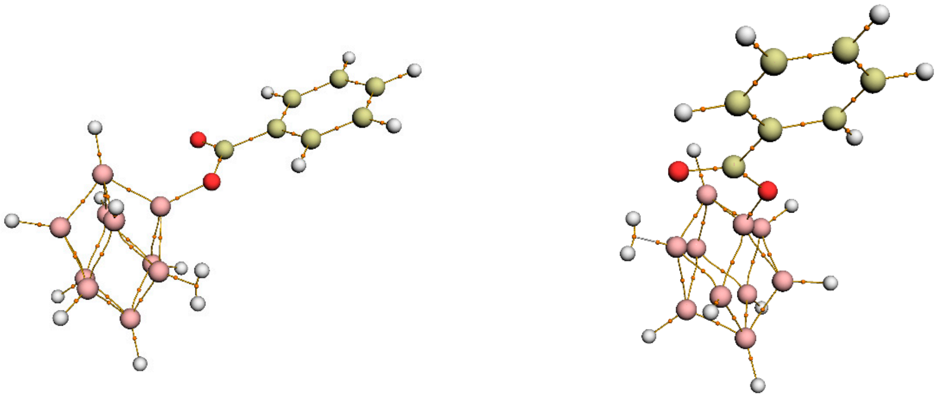

Iso 1 Iso 2

Figure S8. Molecular graph showing the results of the topological analysis of the electron density distribution in the model structure of the $[B_{10}H_8OCOC_6H_5\_H_2]^-$ isomers.

Table S2. Main topological parameters of electron density for B–H_2 interactions. *ρ(r)* – electron density at the bcp, $\nabla^2\rho(r)$ – Laplacian of electron density at the bcp, H_b – total energy at the bcp, δ(B–X) – delocalization index.

	ρ(r) (e Å^{-3})	$\nabla^2\rho(r)$ (e Å^{-5})	H_b (h e^{-1})	δ(B–H)
$[B_{10}H_9\_H_2]^-$	0.126	0.350	-0.097	0.346
$[B_{10}H_8OCOC_6H_5\_H_2]^-$ Iso1	0.126	0.352	-0.098	0.344
$[B_{10}H_8OCOC_6H_5\_H_2]^-$ Iso2	0.127	0.342	-0.099	0.335

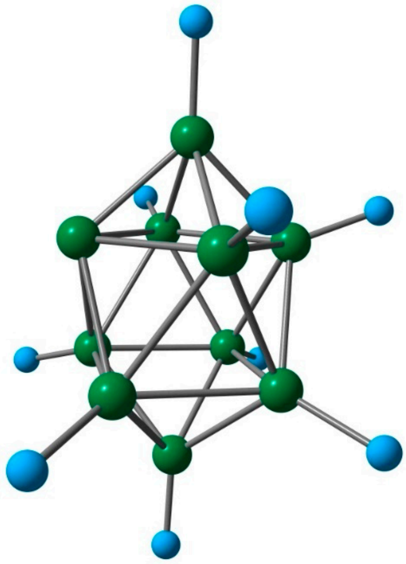

Figure S9. Optimised structure of $[B_{10}H_9]^-$ anion.

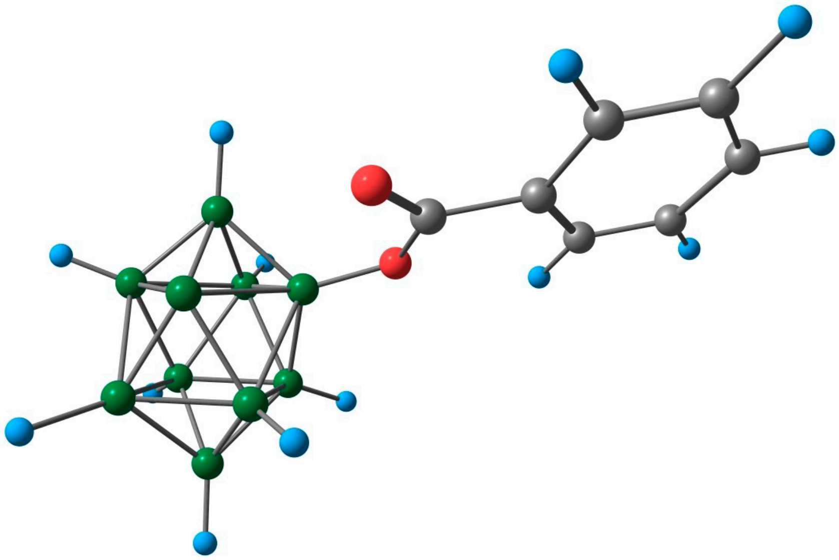

Figure S10. Optimised structure of $[B_{10}H_8OCOC_6H_5]^-$ anion.

Table S3. Cartesian atomic coordinates of the calculated optimized equilibrium model structures. All coordinates are given in Angstrom units.

Chemical species	Atom	x	y	z
$[B_{10}H_{11}]^{1-}$				
	B	-0.015267	1.860433	0.00435
	B	-0.470748	0.787706	-1.205648
	B	1.183959	0.754757	-0.491946
	B	0.506157	0.776805	1.177715
	B	-1.199457	0.737686	0.484847
	H	-0.0528	3.049483	0.024304
	H	-0.930736	1.175585	-2.237457
	H	2.239268	1.114081	-0.922671
	H	0.903821	1.154632	2.238738
	H	-2.242576	1.133446	0.914422
	B	0.462687	-0.736187	-1.198743
	B	0.028602	-1.896334	-0.031207
	B	-1.220644	-0.728746	-0.600021
	B	-0.459644	-0.737482	1.259737

	B	1.160628	-0.744301	0.507131
	H	0.910223	-1.184299	-2.210266
	H	-2.273958	-1.090602	-1.020828
	H	-0.915376	-1.104691	2.296504
	H	2.187264	-1.197481	0.913213
	H	0.133882	-3.07595	-0.08163
	H	-1.172784	-1.752404	0.462288
PhCOOH				
	C	-0.541267	-3.196132	2.867848
	C	-1.621646	-3.688954	3.588916
	C	0.652399	-2.920848	3.513143
	H	-2.555882	-3.902103	3.084238
	H	1.501745	-2.536759	2.963546
	C	-1.506035	-3.909023	4.954904
	C	0.77151	-3.141186	4.882371
	H	-2.348647	-4.291004	5.517244
	C	-0.311157	-3.638702	5.603505
	H	-0.221286	-3.80964	6.667551
	H	-0.629464	-3.028348	1.802036
	C	2.070424	-2.824765	5.533763
	O	3.017224	-2.337172	4.966276
	O	2.09953	-3.134444	6.834462
	H	2.969711	-2.892637	7.179525
$[B_{10}H_{11}]^{1-}$*PhCOOH				
	B	-0.094686	1.882662	0.075418
	B	-0.784572	0.860933	-1.067253
	B	0.978582	0.802464	-0.692545
	B	0.640765	0.750102	1.076889

	B	-1.163243	0.732148	0.726394
	H	-0.124764	3.068886	0.156452
	H	-1.439263	1.292796	-1.967649
	H	1.930136	1.185289	-1.305218
	H	1.237312	1.086002	2.055092
	H	-2.085245	1.138913	1.378516
	B	0.124573	-0.660074	-1.304795
	B	-0.08567	-1.868604	-0.120261
	B	-1.408262	-0.684491	-0.398345
	B	-0.295722	-0.769129	1.287013
	B	1.141413	-0.738849	0.226594
	H	0.360404	-1.063363	-2.402618
	H	-2.520192	-1.03141	-0.638957
	H	-0.540664	-1.172394	2.378455
	H	2.225121	-1.206722	0.400488
	H	0.002675	-3.04491	-0.239617
	H	-1.168291	-1.752418	0.60529
	C	-7.105783	-4.206974	1.631344
	C	-8.159695	-3.512293	1.051002
	C	-5.877524	-3.588633	1.796238
	H	-9.117996	-3.998245	0.914867
	H	-5.046047	-4.121678	2.238726
	C	-7.986409	-2.196306	0.643775
	C	-5.702325	-2.268051	1.392801
	H	-8.806528	-1.655166	0.188968
	C	-6.761838	-1.570548	0.818596
	H	-6.625073	-0.545655	0.501607
	H	-7.241027	-5.232411	1.951204

	C	-4.362679	-1.643688	1.571652
	O	-3.364831	-2.257671	1.868689
	O	-4.365785	-0.327302	1.366943
	H	-3.455266	0.004864	1.438144
TS $[B_{10}H_{11}]^{1-}$				
	B	0.169786	1.816468	-0.009962
	B	-0.281408	0.717409	-1.239031
	B	1.379083	0.723732	-0.510328
	B	0.704755	0.75578	1.16554
	B	-1.07097	0.729126	0.40918
	H	0.125903	3.006721	-0.00217
	H	-0.71353	1.11918	-2.279237
	H	2.430408	1.119247	-0.920193
	H	1.06469	1.113738	2.245401
	H	-2.113611	1.175846	0.784607
	B	0.7008	-0.770493	-1.208858
	B	0.152849	-1.902689	-0.058288
	B	-1.008858	-0.795727	-0.603588
	B	-0.360462	-0.795404	1.097213
	B	1.347165	-0.769364	0.489201
	H	1.173834	-1.224905	-2.208082
	H	-2.045611	-1.187193	-1.049738
	H	-0.777222	-1.438833	2.037108
	H	2.34235	-1.201646	0.988634
	H	0.161191	-3.088714	-0.01135
	H	-1.058556	-0.130118	1.866234
$[B_{10}H_9{*}H_2]^{1-}$				
	B	0.089384	1.815822	-0.335391

	B	-0.343618	0.742494	-1.579588
	B	1.309173	0.731729	-0.808802
	B	0.562875	0.704296	0.846037
	B	-1.131956	0.715179	0.056116
	H	0.079264	3.007132	-0.299514
	H	-0.734036	1.163142	-2.630208
	H	2.369165	1.142701	-1.183987
	H	0.930972	1.052517	1.92912
	H	-2.194029	1.074158	0.472204
	B	0.646676	-0.764999	-1.564617
	B	0.108409	-1.906631	-0.428728
	B	-1.069926	-0.782414	-0.971224
	B	-0.406286	-0.779907	0.68773
	B	1.292464	-0.797132	0.13279
	H	1.122338	-1.169558	-2.585368
	H	-2.098973	-1.187712	-1.421793
	H	-0.811804	-1.65662	1.557343
	H	2.292464	-1.216427	0.633155
	H	0.099316	-3.095458	-0.424301
	H	-0.966248	-0.861047	1.86869
$[B_{10}H_9]^{1-}$				
	B	-0.3018	1.870765	-0.437876
	B	-0.15218	0.654646	-1.677587
	B	1.139427	0.952988	-0.449798
	B	0.014667	0.881994	0.962376
	B	-1.14345	0.507949	-0.267390
	H	2.161972	1.560292	-0.565984
	H	0.017634	1.366836	2.047790

	B	0.95167	-0.697188	-1.221331
	B	0.281016	-1.837925	-0.155572
	B	-0.84415	-0.980837	-1.086241
	B	-0.72837	-0.823196	0.748997
	B	1.067104	-0.540405	0.609088
	H	-1.66423	-1.476995	-1.797234
	H	-1.44716	-1.183512	1.630787
	H	1.951113	-0.761576	1.382721
	H	0.486113	-3.007322	-0.069320
	H	1.734843	-1.055355	-2.050324
	H	-0.2906	0.943864	-2.822270
	H	-0.49219	3.038079	-0.524955
	B	-0.3018	1.870765	-0.437876
	B	-0.15218	0.654646	-1.677587
	B	1.139427	0.952988	-0.449798
	B	0.014667	0.881994	0.962376
	B	-1.14345	0.507949	-0.267390
	H	2.161972	1.560292	-0.565984
	H	0.017634	1.366836	2.047790
$[B_{10}H_9OCOHPh]^{1-}$ iso1				
	O	3.524547	2.564553	9.411584
	O	4.624449	1.819093	7.614702
	C	4.440619	1.887452	8.886669
	C	5.346607	1.119134	9.739865
	C	5.042851	0.957964	11.090893
	H	4.146264	1.403353	11.500221
	C	5.888900	0.216625	11.895488
	H	5.653403	0.080792	12.942732

	C	7.037768	-0.350107	11.357573
	H	7.700304	-0.926208	11.991333
	C	7.343977	-0.181614	10.013036
	H	8.243263	-0.618926	9.599804
	C	6.496945	0.547304	9.197827
	H	6.729899	0.681910	8.150384
	B	0.894358	3.153597	9.295826
	H	0.506683	2.375328	10.111119
	B	2.461552	3.393161	8.733665
	B	1.096531	2.978938	7.618482
	H	0.785487	1.964528	7.065112
	B	0.137751	4.337517	8.370855
	H	-1.043283	4.511383	8.471567
	B	1.522161	4.718225	9.492644
	H	1.581259	5.209109	10.582593
	B	2.54768	3.879526	7.00523
	B	0.895911	4.551363	6.747554
	H	0.196025	4.55964	5.775379
	B	1.195824	5.775327	8.070324
	H	0.744546	6.8671	8.272472
	B	2.862873	5.093062	8.323261
	H	3.884166	5.534578	8.76696
	B	2.280427	5.533201	6.795549
	H	2.725677	6.282477	5.981978
	H	4.000634	2.412137	7.115984
	H	3.278342	3.339513	6.208351
$[B_{10}H_9OCOHPh]^{1-}$ iso2				
	O	3.447391	2.183227	8.693886

	O	1.998425	0.483912	8.681127
	C	3.199784	0.953233	8.730946
	C	4.295409	-0.008076	8.819
	C	5.606412	0.433837	8.643914
	H	5.803536	1.479659	8.450709
	C	6.644649	-0.477385	8.70532
	H	7.663609	-0.143081	8.562376
	C	6.37713	-1.818827	8.948861
	H	7.193131	-2.528911	8.996369
	C	5.071457	-2.256333	9.132552
	H	4.869288	-3.301143	9.327855
	C	4.025514	-1.354369	9.062543
	H	3.006516	-1.688612	9.200941
	B	1.174075	3.383078	9.721015
	H	0.983622	2.730185	10.700503
	B	2.389074	3.248068	8.557026
	B	0.638314	3.138065	8.12213
	H	-0.056425	2.192499	7.857178
	B	0.357797	4.682125	9.026387
	H	-0.620432	5.120389	9.560847
	B	2.126994	4.766483	9.473686
	H	2.709876	5.263362	10.392734
	B	1.875802	3.63372	6.886954
	B	0.422453	4.672779	7.213232
	H	-0.599121	4.823916	6.605464
	B	1.467423	5.825694	8.167774
	H	1.363068	6.997507	8.395574
	B	2.91905	4.789789	7.838528

	H	4.097056	5.004459	7.802836
	B	1.875762	5.300816	6.609359
	H	2.098135	5.91997	5.614426
	H	1.333122	1.209099	8.643498
	H	2.149385	2.83839	6.032749
$[B_{10}H_9OCOPh{*}H^{fac}]^{1-}$ iso1				
	O	3.537621	2.308426	9.469295
	O	5.286733	2.745007	8.12895
	C	4.789073	2.168232	9.069117
	C	5.556997	1.199331	9.913307
	C	5.044355	0.706967	11.110035
	H	4.063028	1.018727	11.440638
	C	5.794177	-0.172342	11.876130
	H	5.394074	-0.546692	12.810065
	C	7.052092	-0.572510	11.445337
	H	7.634157	-1.263666	12.042576
	C	7.564420	-0.086091	10.249036
	H	8.544728	-0.396918	9.910237
	C	6.820291	0.800076	9.487490
	H	7.210305	1.190350	8.556504
	B	1.021650	3.194418	9.592566
	H	0.647142	2.503465	10.485163
	B	2.571078	3.226472	8.887584
	B	1.017579	2.900932	7.940508
	H	0.578070	1.888069	7.487314
	B	0.291153	4.395882	8.641169
	H	-0.848805	4.718339	8.791746
	B	1.755503	4.701868	9.645924

	H	1.964313	5.258809	10.680784
	B	2.484679	3.51467	7.087703
	B	0.943212	4.394995	6.961629
	H	0.196197	4.48084	6.035094
	B	1.470602	5.678193	8.178801
	H	1.162968	6.827691	8.266474
	B	3.063229	4.916842	8.41569
	H	4.137628	5.278289	8.772394
	B	2.387999	5.285042	6.795444
	H	2.711807	5.995591	5.903883
	H	3.483409	4.61837	7.03341
	H	3.105932	2.781577	6.38953
$[B_{10}H_9OCOPh{*}H^{fac}]^{1-}$ iso2				
	O	3.419885	2.136635	8.690272
	O	1.980545	0.450281	8.918177
	C	3.120183	0.856288	8.835161
	C	4.30963	-0.03847	8.869951
	C	5.591736	0.441747	8.617892
	H	5.741512	1.491826	8.406609
	C	6.669116	-0.43048	8.628765
	H	7.665283	-0.0575	8.427386
	C	6.470976	-1.77878	8.894823
	H	7.314625	-2.45793	8.900956
	C	5.192981	-2.25858	9.152515
	H	5.038293	-3.30942	9.362168
	C	4.113945	-1.39059	9.137255
	H	3.112243	-1.75205	9.328985
	B	1.052961	3.377581	9.728098

	H	0.781047	2.900014	10.779099
	B	2.423235	3.164475	8.565471
	B	0.46459	3.199941	8.053953
	H	-0.30305	2.364458	7.692543
	B	0.354325	4.732715	8.972631
	H	-0.5759	5.214032	9.544036
	B	2.132176	4.671709	9.454241
	H	2.664577	5.094194	10.433823
	B	1.845005	3.599643	6.907429
	B	0.456929	4.786867	7.189941
	H	-0.53512	4.986954	6.556328
	B	1.55529	5.815584	8.178871
	H	1.518249	6.978131	8.450427
	B	2.945632	4.717669	7.856398
	H	4.132117	4.845165	7.829476
	B	1.958801	5.270917	6.614541
	H	2.239422	5.845293	5.611699
	H	1.094138	2.31183	8.987303
	H	2.080974	2.81922	6.033247
$[B_{10}H_8OCOPh*H_2]^{1-}$ iso1				
	O	3.651698	2.838859	9.510133
	O	3.55247	3.604265	11.60968
	C	4.033859	2.878885	10.76986
	C	5.155231	1.930633	11.06836
	C	5.670415	1.073445	10.10007
	H	5.254584	1.081257	9.101814
	C	6.711354	0.21325	10.41538
	H	7.108489	-0.45117	9.658159

	C	7.242912	0.204844	11.69784
	H	8.05673	-0.4669	11.94233
	C	6.730184	1.057547	12.66715
	H	7.143587	1.053537	13.66802
	C	5.688826	1.916057	12.35334
	H	5.279688	2.584781	13.09947
	B	0.926849	3.382915	9.392291
	H	0.472349	2.679239	10.23875
	B	2.540712	3.614334	8.976634
	B	1.233681	3.036113	7.764508
	H	1.006685	1.961248	7.295265
	B	0.195712	4.432008	8.283526
	H	-0.99358	4.561923	8.274953
	B	1.468031	4.993989	9.462906
	H	1.389246	5.619173	10.47666
	B	2.662469	3.952975	7.222355
	B	1.051786	4.501655	6.702571
	H	0.458319	4.365997	5.675847
	B	1.22823	5.863417	7.89826
	H	0.751931	6.960354	7.905518
	B	2.876994	5.297643	8.38291
	H	3.859353	5.817423	8.817363
	B	2.42548	5.530344	6.742253
	H	2.925317	6.186706	5.887063
	H	3.596331	3.7108	6.355064
	H	3.436915	2.985605	6.81008
$[B_{10}H_8OCOPh{*}H_2]^{1-}$ iso2				
	O	3.426469	2.169037	8.718629

	O	1.957177	0.489777	8.572864
	C	3.094561	0.897695	8.693483
	C	4.270235	-0.02019	8.810052
	C	5.543857	0.458429	9.104882
	H	5.695176	1.517825	9.26173
	C	6.611258	-0.42168	9.196271
	H	7.600207	-0.04584	9.427338
	C	6.41341	-1.78008	8.986959
	H	7.249769	-2.46511	9.052597
	C	5.143746	-2.26064	8.693029
	H	4.988029	-3.31952	8.528409
	C	4.074783	-1.38308	8.608839
	H	3.080075	-1.74404	8.381531
	B	1.297036	3.549662	9.815098
	H	1.146735	2.990615	10.85246
	B	2.433717	3.224413	8.572527
	B	0.670226	3.252324	8.297784
	H	-0.17384	2.365987	7.837723
	B	0.473405	4.847436	9.080822
	H	-0.44748	5.353943	9.646602
	B	2.277163	4.85326	9.347296
	H	2.941329	5.397272	10.1788
	B	1.760306	3.509982	6.9095
	B	0.352347	4.670019	7.271218
	H	-0.71839	4.796196	6.755637
	B	1.541611	5.832657	8.015675
	H	1.504275	7.022409	8.137325
	B	2.910825	4.693167	7.660643

	H	4.082884	4.857721	7.487738
	B	1.771521	5.141416	6.483064
	H	1.914579	5.650014	5.415435
	H	-0.01513	2.217403	8.677744
	H	1.897552	2.624666	6.119419
TS $[B_{10}H_8OCOPh*H_2]^{1-}$ iso1				
	O	3.724138	3.042867	9.40308
	O	3.482425	3.654424	11.5428
	C	4.035232	3.007096	10.68382
	C	5.177208	2.083319	10.97819
	C	5.76138	1.300001	9.986843
	H	5.384048	1.348267	8.974507
	C	6.82169	0.462081	10.29764
	H	7.273052	-0.14519	9.522962
	C	7.303642	0.403505	11.59823
	H	8.133257	-0.24988	11.83927
	C	6.721431	1.181844	12.59062
	H	7.096573	1.138204	13.60551
	C	5.660244	2.017538	12.28153
	H	5.197431	2.629272	13.04506
	B	1.004424	3.590603	9.308651
	H	0.572441	2.904945	10.18048
	B	2.613701	3.804326	8.86229
	B	1.222709	3.209839	7.676295
	H	0.953056	2.127181	7.252485
	B	0.245624	4.636687	8.21035
	H	-0.93921	4.794463	8.244573
	B	1.565343	5.20154	9.335166

	H	1.521361	5.85005	10.33586
	B	2.688909	4.057617	7.080103
	B	1.045864	4.644679	6.611823
	H	0.431965	4.504327	5.597906
	B	1.296556	6.028005	7.757462
	H	0.859502	7.13981	7.723835
	B	2.939829	5.451303	8.21789
	H	3.946812	5.968195	8.595793
	B	2.461077	5.638847	6.568821
	H	2.91641	6.302777	5.695546
	H	3.437795	4.233538	6.112776
	H	3.470253	3.240856	6.563554
TS $[B_{10}H_8OCOPh*H_2]^{1-}$ iso2				
	O	3.396592	2.07193	8.788371
	O	1.925847	0.389719	8.726745
	C	3.064745	0.799671	8.814601
	C	4.241486	-0.114273	8.945926
	C	5.514764	0.370471	9.231412
	H	5.665525	1.432579	9.368922
	C	6.582530	-0.507374	9.339355
	H	7.571035	-0.127110	9.564456
	C	6.385394	-1.869317	9.153952
	H	7.222026	-2.552756	9.231310
	C	5.115963	-2.355882	8.869027
	H	4.960915	-3.417513	8.722873
	C	4.046255	-1.480811	8.770359
	H	3.051681	-1.846597	8.550767
	B	1.218892	3.394281	9.873320

	H	1.077009	2.837469	10.912581
	B	2.405575	3.128633	8.641713
	B	0.613609	3.152672	8.323617
	H	-0.191433	2.258474	8.022719
	B	0.438923	4.751865	9.170584
	H	-0.489088	5.232539	9.747044
	B	2.226168	4.716781	9.475242
	H	2.862598	5.196822	10.364342
	B	1.777361	3.461209	6.992633
	B	0.339072	4.645756	7.376142
	H	-0.711986	4.806542	6.833389
	B	1.532917	5.757371	8.174593
	H	1.509662	6.941856	8.336606
	B	2.901968	4.621137	7.805989
	H	4.076778	4.790283	7.66447
	B	1.779076	5.10642	6.622504
	H	1.935266	5.639297	5.569306
	H	0.154194	2.285767	9.073619
	H	1.930296	2.619615	6.160445
$[B_{10}H_8OCOPh]^{1-}$ iso2				
	O	3.471199	2.195266	8.746215
	O	1.970995	0.539827	8.859744
	C	3.118807	0.924337	8.843012
	C	4.284750	-0.006983	8.910485
	C	5.586949	0.434267	8.693545
	H	5.772875	1.479977	8.489469
	C	6.638217	-0.469131	8.729815
	H	7.649650	-0.125098	8.554077

	C	6.394635	-1.811227	8.989128
	H	7.217653	-2.514733	9.015863
	C	5.096768	-2.252498	9.214027
	H	4.905585	-3.298243	9.419463
	C	4.044446	-1.352938	9.170501
	H	3.027320	-1.683970	9.334507
	B	1.382263	3.620966	9.920762
	H	1.305215	3.134784	10.999473
	B	2.478703	3.232276	8.628563
	B	0.755136	3.358314	8.453410
	B	0.516949	4.913600	9.177295
	H	-0.343230	5.483958	9.766853
	B	2.331549	4.895113	9.305065
	H	3.038046	5.484010	10.066881
	B	1.631106	3.422295	6.964725
	B	0.266942	4.590123	7.345051
	H	-0.857596	4.669536	6.954587
	B	1.495206	5.809866	7.946846
	H	1.449944	7.003641	7.986672
	B	2.849931	4.637431	7.564191
	H	4.0021	4.779766	7.279762
	B	1.631962	5.030266	6.444751
	H	1.726995	5.498776	5.355065
	H	1.716694	2.453187	6.274307
$[B_{10}H_8O_2CPh]^{1-}$ iso1				
	O	3.647321	2.788108	9.624964
	O	4.299775	3.195018	7.545988
	C	4.495173	2.676157	8.687427

	C	5.732763	1.909793	8.933188
	C	5.896438	1.23829	10.139987
	H	5.11419	1.297083	10.884388
	C	7.047045	0.503446	10.367286
	H	7.172174	-0.02319	11.304789
	C	8.034514	0.441306	9.393602
	H	8.934253	-0.13474	9.572386
	C	7.871713	1.114309	8.190701
	H	8.642232	1.065231	7.431931
	C	6.720988	1.847624	7.956630
	H	6.574843	2.37499	7.023746
	B	0.804312	3.063164	9.219492
	H	0.236234	2.262105	9.894944
	B	2.398027	3.517774	9.122361
	B	1.537565	2.882193	7.687058
	H	1.61583	1.807516	7.161007
	B	0.24959	4.092563	7.983860
	H	-0.91262	4.072359	7.700836
	B	1.113515	4.732321	9.467291
	H	0.729761	5.274579	10.461234
	B	2.971609	3.965041	7.538249
	B	1.45935	4.356503	6.638609
	H	1.117185	4.231398	5.499895
	B	1.168839	5.658824	7.892306
	H	0.567815	6.691097	7.826784
	B	2.692323	5.254969	8.747766
	H	3.477237	5.832358	9.44632
	B	2.646909	5.521338	7.060321

	H	3.25073	6.30245	6.39283
$[B_{10}H_8O_2CPh]^{1-}$ iso2				
	O	2.373506	1.807203	9.153727
	O	0.167133	1.911256	8.780861
	C	1.224567	1.2439	9.066483
	C	1.118974	-0.19929	9.297319
	C	2.256222	-0.93024	9.636789
	H	3.211848	-0.43134	9.721362
	C	2.152022	-2.29088	9.865267
	H	3.032679	-2.86125	10.13044
	C	0.919346	-2.92155	9.753422
	H	0.840723	-3.9862	9.935425
	C	-0.213126	-2.19402	9.409738
	H	-1.171209	-2.68914	9.319892
	C	-0.117511	-0.8324	9.182291
	H	-0.994414	-0.25802	8.916569
	B	1.183169	3.943256	10.06275
	H	0.976778	3.419761	11.11367
	B	2.215901	3.306203	8.875367
	B	0.544251	3.385723	8.59237
	B	0.425554	5.110106	9.151611
	H	-0.431954	5.819669	9.588272
	B	2.307403	5.01772	9.469985
	H	3.040734	5.647422	10.17389
	B	1.645828	3.434947	7.182659
	B	0.345115	4.676593	7.367389
	H	-0.690381	4.871899	6.799753
	B	1.628417	5.836853	7.987355

	H	1.688197	7.033412	7.981862
	B	2.926219	4.548775	7.802707
	H	4.104315	4.635228	7.609518
	B	1.825796	4.972634	6.560464
	H	2.02971	5.343259	5.445849
	H	1.699686	2.414212	6.560183
